# The mimetic wing pattern of *Papilio polytes* butterflies is regulated by a *doublesex*-orchestrated gene network

**DOI:** 10.1038/s42003-019-0510-7

**Published:** 2019-07-10

**Authors:** Takuro Iijima, Shinichi Yoda, Haruhiko Fujiwara

**Affiliations:** Department of Integrated Biosciences, Graduate School of Frontier Sciences, The University of Tokyo, Kashiwa, Chiba, 277-8562 Japan

**Keywords:** Gene regulation, Mimicry, Transcriptomics, RNAi

## Abstract

The swallowtail butterfly *Papilio polytes* is sexually dimorphic and exhibits female-limited Batesian mimicry. This species also has two female forms, a non-mimetic form with male-like wing patterns, and a mimetic form resembling an unpalatable model, *Pachliopta aristolochiae*. The mimicry locus *H* constitutes a dimorphic Mendelian ‘supergene’, including a transcription factor gene *doublesex* (*dsx*). However, how the mimetic-type *dsx* (*dsx-H*) orchestrates the downstream gene network and causes the mimetic traits remains unclear. Here we performed RNA-seq-based gene screening and found that *Wnt1* and *Wnt6* are up-regulated by *dsx-H* during the early pupal stage and are involved in the red/white pigmentation and patterning of mimetic female wings. In contrast, a homeobox gene *abdominal-A* is repressed by *dsx-H* and involved in the non-mimetic colouration pattern. These findings suggest that dual regulation by *dsx-H*, induction of mimetic gene networks and repression of non-mimetic gene networks, is essential for the switch from non-mimetic to mimetic pattern in mimetic female wings.

## Abstract (web summary)

Takuro Iijima, Shinichi Yoda, and Haruhiko Fujiwara investigate gene networks controlled by the mimicry-associated allele of *doublesex*, *dsx-H*, in butterflies that mimic an unpalatable species. They find that *dsx-H* has a dual function: it induces mimetic gene networks and represses non-mimetic gene networks, explaining its key role in the switch between mimetic and non-mimetic female forms.

## Introduction

Mimicry, by which mimetic species trick predators by resembling another species, is a widespread survival strategy used by many animals and which has long been studied in various academic fields, such as ecology, animal behaviour and evolutionary biology^1^. To avoid predation, non-toxic palatable species often evolved to resemble a distantly related unpalatable model species in shape, colour patterns or behaviour^2^, a phenomenon called Batesian mimicry^3^. Many butterflies exhibit Batesian mimicry; in some species, only the females resemble the model butterfly, a form known as female-limited Batesian mimicry^4^. This phenomenon has attracted not only researchers but also the public since the time of Darwin^1^.

**Fig. 1:**
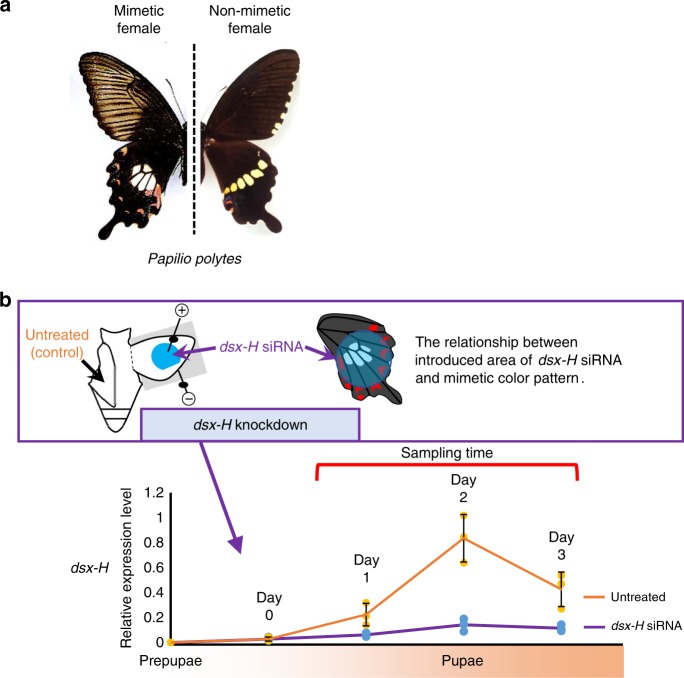
Schematic view of analysis of differentially expressed (DE) genes by *dsx*-*H* knock-down. **a** Adult wing-colour patterns of non-mimetic female and mimetic female of *Papilio polytes*. **b** Scheme of sampling for RNA-Seq samples. *dsx-H* siRNA was injected into the left pupal hindwing immediately after pupation and electroporated into the ventral side (schematic at upper panel). We estimated the ratio of *dsx-H* reduction between untreated (orange bar) and treated wings (purple bar) by siRNA in the same individual by real-time PCR using *RpL3* as an internal control. Values and error bars denote the mean and standard deviation of three biological replicates

The common Mormon swallowtail butterfly, *Papilio polytes* L., is widely distributed in southeastern to southern Asia, as well as in Okinawa in Japan. *P. polytes* is sexually dimorphic, exhibits female-limited Batesian mimicry and has two female forms; a certain proportion of the female population (‘mimetic female’) mimics an unpalatable (to bird predators of swallowtail butterflies) model butterfly, the common rose *Pachliopta aristolochiae*^5,6^ (Fig. 1a). In the hindwings of the mimetic female, we observe a white pigmented area in the centre and red spot markings at the wing margins. On the other hand, non-mimetic females and all males (which are non-mimetic) show a white band pattern across the hindwings (Fig. 1a). Several genetic studies have revealed that the mimetic phenotype of *P. polytes* is controlled by a single autosomal *H* locus^7^, and that the mimetic trait (*H*) is dominant to the non-mimetic trait (*h*)^8^. It is noteworthy that females with a *HH* or *Hh* genotype show mimetic wing colour and pattern traits; however, males, even with the *HH* or *Hh* genotype, show wing colour and pattern traits similar to those of non-mimetic females (*hh*). The *H* locus of *P. polytes* affects not only the wing colouration pattern but also the shape of the wing^9^ and flight behaviour^2^. It has been hypothesised that the polymorphic and complex adaptive traits, which are involved in creating the mimic phenotype of *P. polytes*, are controlled by multiple genes at a single chromosomal locus that behave together as a ‘supergene’^10,11^. The supergene hypothesis has been proposed for many adaptive traits in various species: Batesian mimicry in *Papilio dardanus*^12,13^, Müllerian mimicry in *Heliconius numata*^14^, bird feather polymorphism, fish cryptic pattern polymorphism and the heteromorphic self-incompatibility floral structures in plants such as primrose^15,16^. Although the chromosomal location and structure responsible for the *P. polytes* supergene has been revealed recently, the detailed molecular mechanism underlying each supergene phenomenon remains unclear in most cases still.

Recent studies on Batesian mimicry of *P. polytes* have clarified the responsible region for the *H* locus near the transcription factor (TF) gene *doublesex* (*dsx*) on chromosome 25^9,17^. There are two types of tightly linked cluster of loci on chromosome 25, which are associated with dimorphic alleles: mimetic (*H* allele) and non-mimetic (*h* allele). The region responsible for the *H* locus is about 130 kb long, includes *dsx* and two additional genes, *ubiquitously expressed transcript* (*UXT*) and a long noncoding RNA gene (*U3X*). The *H* locus region is highly diversified in sequence and inverted outside the entire *dsx* gene between the *H* and *h* allele. In addition, functional analysis using in vivo electroporation-mediated RNA interference (RNAi) has revealed that knock-down of the mimetic-type *dsx* (*dsx-H*) but not the non-mimetic-type *dsx* (*dsx*-*h*) switches the mimetic colouration pattern to the non-mimetic colouration pattern in the hindwings of mimetic females^17^. This indicates that Dsx-H is a key factor in inducing the mimetic phenotype and repressing the non-mimetic phenotype; however, it is largely unknown how this TF orchestrates the downstream gene network in Batesian mimicry.

It is known that RNAi is usually ineffective among the Lepidoptera^18^, and thus, in vivo electroporation-mediated small interfering RNA (siRNA) incorporation into the lepidopteran cells results in a mosaic area^17,19^. In the current study, we take advantage of this situation (mosaic RNAi) to compare the transcriptome between siRNA-treated and untreated area in wings of the same pupa. Using this method, we knocked-down *dsx-H* in early pupal wings of *P*. *polytes* mimetic females, performed comparative RNA sequencing (RNA-Seq) analyses, and succeeded in drawing-up a comprehensive list of target genes controlled by *dsx-H* during the pre-patterning process of mimetic colouration. We selected three key genes, *Wnt1, Wnt6*, and *abd-A*, for further functional analysis, and demonstrated that *Wnt1* and *Wnt6*, up-regulated by *dsx-H*, were involved in the mimetic colouration and patterning, while *abd-A*, down-regulated by *dsx-H*, was involved in controlling the non-mimetic colouration and patterning. We discuss further how control of expression of these genes occurs in the pre-patterning process and how the switch from non-mimetic to mimetic wing colouration patterns occurs.

## Results

### Screening of the genes involved in the mimetic gene networks downstream of *dsx-H*

To screen the genes or gene networks involved in the *P. polytes* mimetic wing colouration and patterning, which are controlled by the mimetic *dsx-H* function, RNA-Seq analysis is a practical and effective method. It is possible to compare the RNA-Seq data between mimetic and non-mimetic female wings; however, such a comparison often results in false positives due to differences between individual butterflies. To overcome this problem, we established a novel screening method to our knowledge, using in vivo electroporation-mediated RNAi, which enables us to knock-down expression of the target gene only near the positive (+) electrode or anode (Fig. 1b). After knocking-down *dsx-H* in the mimetic female using this method, we have previously succeeded in changing the wing colouration from the mimetic to the non-mimetic pattern^17^.

**Fig. 2:**
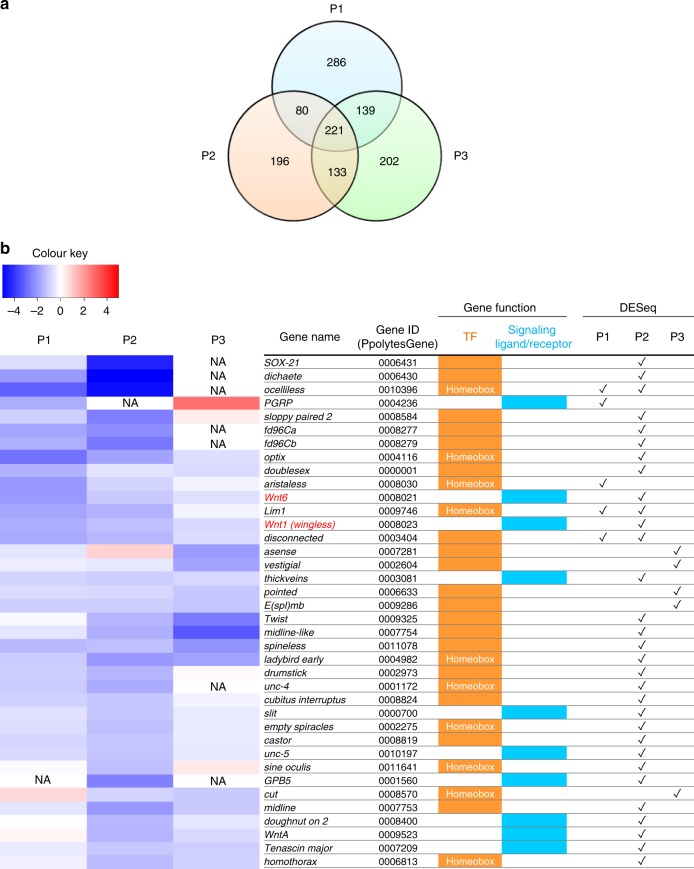
Details of DE genes identified by *dsx-H* knock-down. **a** Venn diagram depicting the abundance of DE genes (*P* < 0.05) for each comparison between wing sampling stages by untreated and siRNA-treated samples. **b** Heatmap of DE genes down-regulated by *dsx-H* knock-down. The colour key indicates scaled log-two-fold change value (Log2-fold change value = Log2 siRNA-treated FPKM–Log2 untreated FPKM). Bars coloured orange or blue in the Gene function column represent transcription factor and signalling molecules, respectively. Check marks in the DESeq column indicate *P* < 0.05 in the DESeq analysis. NA not applicable (=missing value)

First, we introduced siRNA for *dsx-H* by electroporation into a hindwing of a mimetic female immediately after pupal ecdysis. Next, after 1–3 days (P1–P3, respectively), RNA samples were prepared each day from the *dsx-H* knocked-down wing and from another, untreated wing in the same pupa (Fig. 1b). By quantitative real-time-polymerase chain reaction (qRT-PCR), we confirmed that *dsx-H* expression was repressed by siRNA treatment in the knock-down wings (Fig. 1b, bottom figure). It is noteworthy that we can compare the transcriptome between *dsx*-knocked-down and normally developed wings in the same individual at the same developmental timing by using this novel method. Using RNA samples in which the expression levels for *dsx-H* had been confirmed, as mentioned above, a total of six libraries (siRNA knocked-down and untreated wings for periods P1–P3) were constructed and used for the RNA-Seq analyses (Supplementary Table 1). RNA-seq-based screening in this study has an exploratory value, since each comparison consists of one biological replicate. The read data obtained were mapped to the Ppolytes.v1.0.0. transcriptome^17^ using the analytical sequence alignment software Bowtie 2^20^. Finally, comparing the gene expression levels from the mapping situation by using the analytical software DESeq^21^, we searched for genes in which expression had changed between normal and *dsx-H*-knocked-down samples, and obtained 726, 630 and 695 genes at the P1, P2 and P3 stages, respectively (Fig. 2a and Supplementary Fig. 1). Among these differentially expressed (DE) genes, there were 49, 109 and 54 genes down-regulated by *dsx-H*-knock-down at stages P1–P3, respectively, while up-regulated genes were 677, 521 and 641 at P1–P3, respectively (Supplementary Fig. 1). Expression of a total of 221 genes changed similarly between normal and *dsx-H*-knocked-down samples through stages P1–P3 (Fig. 2a).

Since most of the genes screened for above are uncharacterised (designated unannotated or unknown), we further screened the genes characterised so far to review their potential functional roles in the gene regulatory network of mimetic pattern formation. We here focused on genes encoding either TF or signalling molecule (ligand or receptor), because they mainly regulate the gene network involved in various developmental events. Figure 2b shows a heatmap of down-regulated genes including 28 TFs and 10 signalling molecules that were picked up either at P1, P2 or P3 stage (Supplementary Fig. 1) based on DESeq results; in contrast, Supplementary Fig. 2 shows a heatmap of up-regulated genes including 21 TFs and 24 signalling molecules. Our previous report suggested that *dsx-H* not only induces the mimetic pattern but also represses the non-mimetic pattern in female hindwings^17^. Therefore, genes whose expression is decreased by *dsx-H* knock-down are thought to be positively regulated by *dsx-H*, and possibly involved in the mimetic pattern formation. In contrast, genes whose expression is increased by *dsx-H* knock-down are thought to be negatively regulated by *dsx-H*, and possibly involved in the non-mimetic pattern formation. We here succeeded in detecting many TFs and signalling molecules effectively, probably because the transcriptome between *dsx-H* knocked down and control samples was compared in the same individual at the same developmental timing. Especially, it is remarkable that 10 TFs encoding homeobox genes (*cut, optix, aristaless, Lim1, empty spiracles, homothorax, ladybird early, sine oculis, ocelliless, unc-4*) are included in the list of down-regulated genes (Fig. 2b). Homeobox genes such as *Distal-less*, *engrailed* and *spalt* are involved in the eyespot formation in *Bicyclus anynana*^22–24^, suggesting that some homeobox genes found in this study may play pivotal roles in inducing the colour pattern formation in the mimetic wings. It is of great interest that major wing patterning genes in *Heliconius* butterflies, *optix*, *aristaless* and *WntA* are also included in the list. It is known that *optix* and *aristaless* are homeobox genes involved in determining the red wing pattern in *H. erato*^25^ and in controlling the colour change of yellow/white pattern in *H. cydno*^26^, respectively. *WntA* is a morphogen determining the black pattern of the forewing in *H. erato*^27^, suggesting that *Heliconius* wing patterning genes are potential candidates for controlling mimetic pattern in *P. polytes*. Regarding signalling molecules up-regulated by Dsx-H, we found Wnt-signalling ligands, *Wnt1 (wingless)* and *Wnt6*, and a receptor for Decapentaplegic (Dpp), *thickveins*, which are suggested to be involved in the colour pattern formation in some nymphalid butterflies^22,28^ (Fig. 2b). However, it is noteworthy that we found no gene related to wing patterning in the list of down-regulated gene by Dsx-H (up-regulated by *dsx-H* knock-down) (Supplementary Fig. 2). As down-regulated genes, a hox gene *abdominal-A* (*abd-A*), some ecdysone-signalling genes *broad*, *E74* and *hormone receptor 3* (Supplementary Fig. 2) are listed. Previous studies on the wing colouration have shown that hox genes, such as *Ultrabithorax* and *Antennapedia* are involved in the eyespot formation in *B. anynana*^29,30^, and that *ecdysone receptor* (*EcR*) regulates the eyespot development in some butterflies^31^, suggesting that *abd-A* and the ecdysone-signalling gene network may have some important roles in regulating non-mimetic pattern in *P. polytes* wings.

### Colour pattern-associated expressions of *Wnt1, Wnt6* and *abd-A* under controls of *dsx-H*

Of the genes in the above lists, we focused here on two genes, *Wnt1* and *Wnt6*, the expression of which was repressed by *dsx-H* knock-down at P2 (Fig. 2b) and on *abd-A*, the expression of which was induced by *dsx-H* knock-down at P1 (Supplementary Fig. 2), since they are essential genes for various patterning processes and have been shown be involved in the formation of other colour patterns. *Wnt1* and *Wnt6* belong to the *Wnt* family (Supplementary Fig. 3), which are known to act as morphogens and to play an important role in appendage/wing development^32,33^, the wing colouration in Nymphalidae^28^ and the larval spot pattern formation in *Bombyx mori*^34^. The homeobox gene *abd-A* is involved in thoracic and abdominal formation^35^. It is reported that *dsx* controls male-specific abdominal colouration through the *Abd-B* function^36^; however, to date, the involvement of *abd-A* in colour pattern formation has not been reported in Lepidoptera.

**Fig. 3:**
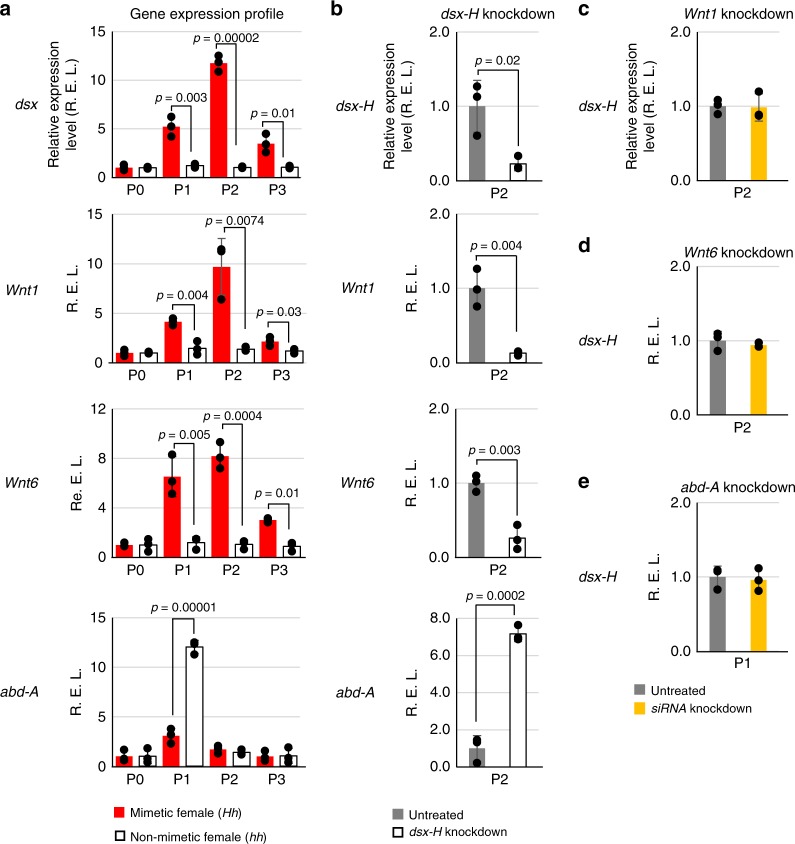
The expression levels of three genes (*Wnt1, Wnt6* and *abd-A*) controlled by *dsx-H* in *P. polytes*. **a** Expression changes over time (P0–P3) of four genes (*dsx, Wnt1, Wnt6* and *abd-A*) in mimetic female (*Hh*) and non-mimetic female (*hh*). The expression levels of all genes are shown as relative values with the expression level of P0 taken as 1. **b** Relative expression levels of four genes in the *dsx-H* knock-down wing of mimetic female (*Hh*) in P1 and P2. The expression levels are shown as relative values with the expression level in the untreated wing of mimetic female (*Hh*) taken as 1. **c**–**e** Relative expression level of *dsx-H* in three gene (*Wnt1, Wnt6* and *abd-A*) knock-downs at P1 and P2. The expression levels are shown as relative values with the expression level in the untreated wing of mimetic female (*Hh*) taken as 1. We estimated the gene expression levels by real-time PCR using *RpL3* as the internal control. Error bars show standard deviation of three biological replicates. *P < 0.05 for Student’s *t-*test

To determine the exact expression profile of these genes at the early pupal stages, when mimetic pre-patterning is presumably determined, we quantified mRNA levels of four genes (*dsx*, *Wnt1*, *Wnt6*, *abd-A*) by qPCR (Fig. 3a). Consistent with the results in Fig. 1b and from our previous report^17^, expression of the *dsx-H* gene peaked at P2 in *Hh* females (Fig. 3a, red column) but was not induced in non-mimetic *hh* females (Fig. 3a, open column). Similar to *dsx-H*, expression of *Wnt1* and *Wnt6* peaked at P2 in mimetic females (Fig. 3a, red column), but was not induced in non-mimetic females (Fig. 3a, open column), suggesting that both genes are rapidly induced by *dsx-H* but only in mimetic females. In contrast, *abd-A* expression was observed primarily at P1 in non-mimetic females (Fig. 3a, open column) but less so in mimetic females (Fig. 3a, red column), which suggests that *dsx-H* immediately represses *abd-A* expression. We had reported previously that *dsx-h* is also expressed at about 50% of the level of *dsx-H* at P2 in *Hh* mimetic females^17^. Combining this observation with the present results, that *Wnt1/Wnt6* are little expressed during P1–P3 while *abd-A* is highly expressed at P1 in non-mimetic *hh* females (Fig. 3a), we speculate that only *dsx-H* can induce *Wnt1/Wnt6* and repress *abd-A*, while *dsx-h* does not have any such regulatory functions.

Next, to determine whether *dsx-H* induced *Wnt1/Wnt6* and repressed *abd-A*, we quantified the expression of each gene by qPCR after knocking-down *dsx-H* in the *Hh* mimetic female pupa. Using the same RNA samples in which *dsx-H* expression was clearly repressed in the wing to which siRNA was introduced (Fig. 3b, top, *dsx-H* knock-down), relative to the untreated wing (Fig. 3b, top, untreated), we found that *Wnt1/Wnt6* expression decreased and *abd-A* expression markedly increased (Fig. 3b, *Wnt1, Wnt6, abd-A*). Contrary to this, when the expression of any of *Wnt1, Wnt6* and *abd-A* was knocked-down, the expression level of *dsx-H* did not change between the knocked-down and untreated wings (Fig. 3c–e). These analyses clearly show that *Wnt1*, *Wnt6* are positively and *abd-A* is negatively regulated as downstream genes by *dsx-H*. To know whether there is a possible genetic interaction between *Wnt1/Wnt6* and *abd-A*, we quantified by qPCR the *abd-A* expression in cDNA samples of *Wnt1* or *Wnt6* knockdown (Supplementary Fig. 4). We found, however, no significant difference in the expression level between siRNA treated and non-treated wings in both cases (Supplementary Fig. 4). This suggests that up-regulation of *abd-A* is caused by the *dsx-H* down-expression but not through the *Wnt1*/*Wnt6* down-regulation.

Focusing on the colour pattern of *P. polytes* hindwings, especially with respect to red or white (or pale yellow) regions, we found that the mimetic pattern changes depended on the female genotype (Supplementary Fig. 5), at least in the lab-bred Japanese population. In the *HH* female hindwings, the central white region was replaced by the red pattern and the peripheral red spots became larger, in comparison with the *Hh* female wings (Supplementary Fig. 5a). By measuring the area of the colouration region using the Image J programme, it was shown that the area of the red region (expressed as % of total hindwing area) in the *HH* mimetic females was larger than that in the *Hh* females (Supplementary Fig. 5b), while the area of the white region showed the reverse tendency (Supplementary Fig. 5c). Meanwhile, in the non-mimetic females and males, all of which showed white banding patterns on the hindwings, the area of the white region did not differ between the males and females (Supplementary Fig. 5d, e). It is noteworthy that the level of expression of *dsx-H* in male wings seemed very low during the pupal stages^17^, suggesting that males of any genotype (*HH*, *Hh*, *hh*) showed a wing pattern similar to each other and to non-mimetic *hh* females. Combining this fact with the hypothesis that knock-down of *dsx-H* in mimetic female wings caused the change from mimetic to non-mimetic colouration pattern, it is assumed that *dsx-H* mainly controls the above dosage effect of *H* on the area of the red and white regions on the wings of females.

To determine whether the size of the area of red or white colouration is controlled via *Wnt1*/*Wnt6* induced by *dsx-H*, next, we investigated their colour-pattern-associated expression. After each coloured area of hindwings at P2 (future red, black or white) was cut, RNA samples were prepared. Using qPCR, we found that *Wnt1* was expressed in both the white and red regions but *Wnt6* was expressed mainly in the red spots in a region-specific manner in the mimetic female (Supplementary Fig. 6). However, these expression patterns were not observed in non-mimetic *hh* females. *Wnt1* expression in the red region was higher in *HH* female individuals than in *Hh* females (Supplementary Fig. 6b, c), suggesting that the above dosage effect of *H* on the area of the red region was controlled via *Wnt1*. Higher expression of *Wnt1* in the white region may cause the change from white to red.

### Functional roles of *Wnt1*, *Wnt6* and *abd-A* on wing colouration

**Fig. 4:**
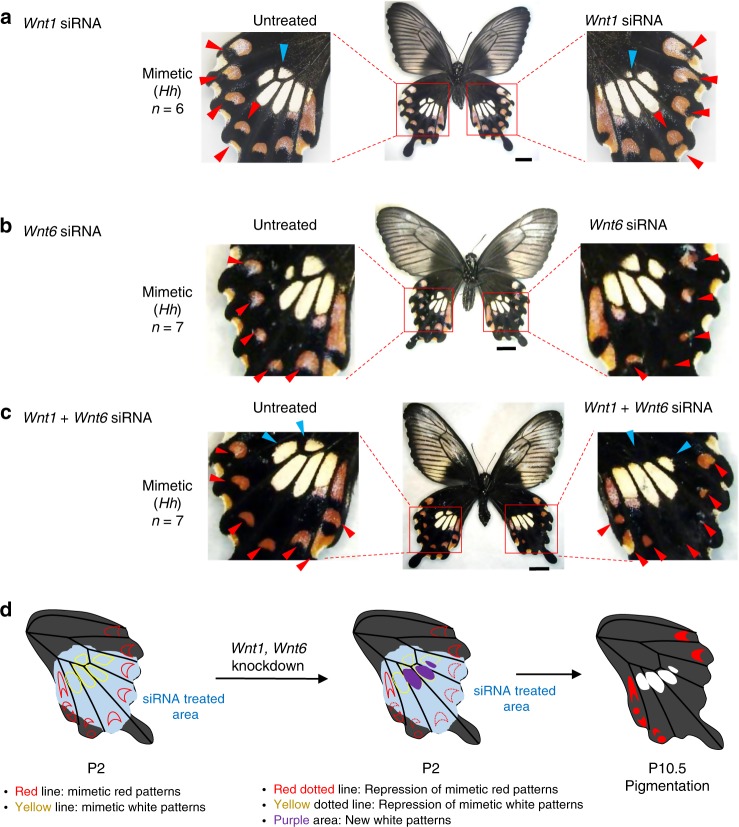
Knock-down of *Wnt-*signalling pathway genes in hindwings of mimetic female of *P. polytes*. **a**–**c** The phenotypes of mimetic females with knock-down of *Wnt1* (**a**), *Wnt6* (**b**) and *Wnt1* and *Wnt6* (**c**). Each siRNA was injected into the left pupal hindwing immediately after pupation and electroporated into the ventral side. Red and blue arrowheads represent the changed red and white regions, respectively. Scale bars, 1 cm. Supplementary Figs. 8–10 show other replicates. **d** The effect model of the knock-down of *Wnt1* and *Wnt6* in the hindwing

**Fig. 5:**
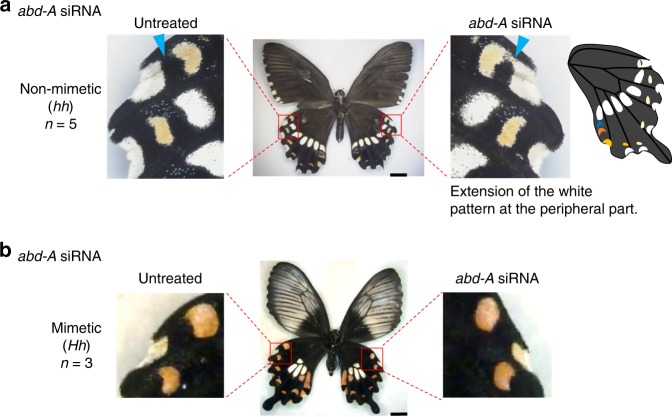
Knock-down of *abd-A* in mimetic female hindwings of *P. polytes*. **a**, **b** The phenotypes of mimetic females with knock-down of *abd-A* in non-mimetic (**a**) and mimetic female (**b**). Each siRNA was injected into the left pupal hindwing immediately after pupation and electroporated into the ventral side. Blue arrowheads represent the extended white regions (schematic at right). Scale bars, 1 cm. Supplementary Figs. 11 and 12 show other replicates

To understand the functional roles of *Wnt1/6* and *abd-A* on wing colouration patterns better, we performed in vivo electroporation-mediated RNAi (Figs. 4 and 5). After injecting siRNA for each gene into one hindwing of a pupa immediately after pupation, we performed electroporation and observed the phenotypic changes in the adult stage. After the knock-down treatment, we quantified mRNA expression of each gene at P1 and P2 and determined that the expression level was significantly decreased only in the siRNA-treated wing (Supplementary Fig. 7). When *Wnt1* was knocked-down in mimetic female wings, peripheral red spots changed its shape from dotted to extended pattern toward the wing margin (Fig. 4a and Supplementary Fig. 8, red arrowhead). In some *Wnt1* knocked-down samples, we observed a drastic expansion of peripheral red spots which merged with pigments on the wing margin (Supplementary Fig. 8, red arrowhead), whereas the central white patch was shrunken in size (Fig. 4a and Supplementary Fig. 8, blue arrowhead). These phenotypic changes indicate that *Wnt1* has a dual role across mimetic hindwing: the one for controlling the shape of red spots on wing margin, and the other for inducing the white patch on wing central part. When *Wnt6* was knocked-down, the red spot pattern shrank compared with the non-treated wing, but no change was observed in the white pattern in the central part (Fig. 4b and Supplementary Fig. 9). These data suggest that *Wnt1* and *Wnt6* are functionally differentiated in the formation of red and white colouration patterns. Next, we knocked-down both *Wnt1* and *Wnt6* (i.e. double knock-down) in mimetic female wings and observed clearly shrunken peripheral red spots (Fig. 4c and Supplementary Fig. 10), some of which merged with patches on the wing margin (Supplementary Fig. 10), and the disappearance of the central white spot (Fig. 4c and Supplementary Fig. 10). As compared to untreated wings showing the mimetic phenotype, the knocked-down wing showed similarity with the non-mimetic pattern, with a linearised edge of the central white region and lacking peripheral red spots. We had shown previously that knock-down of *dsx-H* in mimetic female wings caused the mosaic change (or partial reversal effects) to non-mimetic wing phenotype^17^. Therefore, some part of switching from mimetic to non-mimetic wing patterns is mediated via *Wnt1*/*Wnt6*. However, the non-mimetic hindwing shows a more extended (and band-shaped) white region; some other factors controlled by *dsx-H*, as shown in the gene list in Fig. 2, appear to be necessary for the complete switching from mimetic to non-mimetic. Further functional analyses of genes in the list in Fig. 2, will clarify this possibility (Fig. 4d).

The above data suggested that *abd-A* is involved in controlling the non-mimetic wing pattern; therefore, next, we performed *abd-A* knock-down in both mimetic and non-mimetic female wings and found that the phenotypic changes occurred only in the latter wings: the white spot in the margin of the wing extended to fuse with the flanking spots in non-mimetic females (Fig. 5a and Supplementary Fig. 11). However, no phenotypic change was observed in the hindwings of mimetic females (Fig. 5b and Supplementary Fig. 12). Although the contribution to the non-mimetic pattern formation seems to be not particularly large, we think that *abd-A* is involved in part of non-mimetic patterning. Importantly, the same phenotypic change achieved by *abd-A* knock-down was also observed in males (Supplementary Fig. 13). In addition to *abd-A*, it was suggested that *dsx-H* repressed many genes (Supplementary Fig. 2), some of which may be involved in the formation of non-mimetic wing colouration. Previous studies in *Drosophila* revealed that *abd-A* is expressed in the thorax and abdominal regions and involved in their formation^35^, and in switching the male-specific abdominal colouration by regulating *dsx* expression^36^. In *P. polytes*, conversely *abd-A* is controlled by *dsx* directly or indirectly, and it is not clear yet whether there are similar gene networks, causing colour pattern formation, between *P. polytes* and *D. melanogaster*.

### Dsx-H drives gene networks causing mimetic wing colouration patterns

**Fig. 6:**
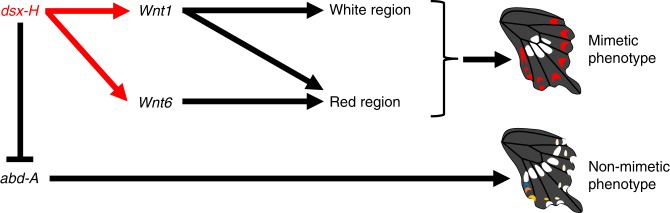
Model for *dsx-H, Wnt1, Wnt6* and *abd-A* functions in regulation of wing-pattern formation. The gene cascade of mimetic pattern formation in mimetic female hindwing was predicted from expression and functional analyses

The present study has shown that the colour pattern switch from non-mimetic to mimetic pattern controlled by Dsx-H involves not only the induction of mimetic gene networks, including *Wnt1* and *Wnt6*, but also the repression of non-mimetic gene networks, including *abd-A* (Fig. 6). It is assumed that chromosome 25 (Chr. 25) carrying *dsx-H* originated from Chr. 25 carrying *dsx-h*, based on comparison of the amino-acid sequences of Dsx among Lepidoptera and on the orientation of the chromosomal inversion outside the *dsx* locus^9,17^. Thus, the mimetic wing colouration pattern might have evolved from the non-mimetic one, although we do not know how *dsx-H* evolved to orchestrate gene networks to induce the mimetic pattern and to repress the non-mimetic pattern. The above results show that *abd-A* is involved in part of the non-mimetic pattern formation (Fig. 5) and the expression of *abd-A* is repressed by *dsx-H* at P1 (Fig. 3a). In a similar way, *dsx-H* may suppress the expression of key TFs other than *abd-A* and signalling molecules, which leads to the overall repression of non-mimetic colouration patterns, although further studies will be needed to test this hypothesis. Genetic mapping and association studies in some butterflies have shown that only a small subset of genes, such as *dsx*, *optix*, *WntA*, *cortex* and *aristaless* play a causative role in the wing pattern formation. All of these genes have been genetically associated with the local adaptation in multiple populations, and may have been co-opted into gene regulatory networks that control the wing pattern formation. As in the cases of *dsx* in *P. polytes*^9,17^ and *P. memnon*^37^, *cortex* in *Heliconius*^38^ and the peppered moth^39^, *WntA* and *optix* in some nymphalid butterflies^25,27,40^, a small set of genes control the genetic variation generating the wing pattern diversity across different lineages. It is hypothesised that they can function as adaptive ‘hotspot’ genes that have repeatedly caused the evolution of similar traits in independent lineages^41^. There is a possibility that both convergent and divergent evolution of a great diversity of wing patterns is controlled by just a few hotspot genes^42^. This study takes a bold step toward understanding the details of downstream gene network of the potential hotspot gene, *dsx*, in *P. polytes*.

We propose two models with which to explain the functional difference between Dsx-H and Dsx-h in association with the mimetic pattern formation. The first model is the functional difference depends on a difference in the protein function between them, since there are 15 amino-acid differences between Dsx-H and Dsx-h in *P. polytes*. We have shown previously that *dsx-H*-specific siRNA, but not *dsx-h*-specific siRNA, could switch the colour pattern in the mimetic female wings shortly after pupal ecdysis^17^. However, expression of *dsx-h* is repressed to a lower level in pupal wings of *Hh* mimetic females and, thus, the knock-down of *dsx-h* may be primarily ineffective. In addition, a recent study on female-limited Batesian mimicry of a closely related species, *Papilio memnon*, revealed that the sites where amino acids changed in the amino-acid sequence between mimetic Dsx (Dsx-A in *P. memnon* and Dsx-H in *P. polytes*) and non-mimetic Dsx (Dsx-a in *P. memnon* and Dsx-h in *P. polytes*) were totally different between the two butterfly species, indicating that there was no conserved amino-acid specifically involved in the mimetic phenotype^37^. The other model is that the mimetic phenotype depends on higher expression level of *dsx* independently of the amino acid sequence differences between Dsx-H and Dsx-h: during the early pupal stages, high levels of *dsx* (*dsx-H*) expression in *HH* or *Hh* females may cause the mimetic phenotype, whereas low levels of *dsx* (*dsx-h*) expression in *hh* female may cause the non-mimetic phenotype. It is noteworthy that peak expression of *dsx-H* at P2 is only observed in female wings (mimetic) but not in male wings (non-mimetic), which explains female-limited mimicry convincingly. This indicates that some *cis*-regulatory elements which increase transcription in a female-specific manner have accumulated at the *dsx-H* locus during evolution. Further experiments studying the effect of over-expression of *dsx-H* or *dsx-h* in wings of non-mimetic females (or males) will clarify which model is the more appropriate.

In some Nymphalidae butterflies, pre-patterning of adult colour patterns are suggested to occur, mainly during the pre-pupal stages^43^. However, the current study reveals that pre-patterning of *P. polytes* wing colouration is not completed even by pupal ecdysis, because knock-down of not only *dsx-H* but also *Wnt1*, *Wnt6* and *abd-A* immediately after ecdysis could change the adult wing patterns. Since knock-down of *Wnt1*, *Wnt6* and *abd-A* resulted in the loss or change in size of the pigmented area, it is suggested that these genes are involved in the regulation of pigment synthesis. In the mimetic female wings, we had suggested previously that the peripheral red pigments are composed of N-β-alanyldopamine (NBAD) and kynurenine, but that the white pigments in the centre do not include kynurenine^44^. In addition, the related genes involved in biosynthesis of these compounds are highly expressed in a region-specific manner at the late pupal stages^43^. The above results suggest that both *Wnt1* and *Wnt6* control genes that are involved in the synthesis of peripheral red pigments, while *Wnt1* also regulates synthesis of the white pigment in the centre. Thus, it is possible that only *Wnt6* is involved in a major way in the kynurenine synthesis pathway.

Importantly, by knocking-down *Wnt1/Wnt6*, the shape of the centred white area changed from the mimetic to the non-mimetic pattern, suggesting that these *Wnt* genes control spatial expression of genes related to pigment synthesis in the hindwings. Recently, we have reported that the female-limited Batesian mimicry in *P. polytes* and *P. memnon* is caused by the almost-identical chromosomal locus (*H* and *A*) on Chromosome 25^37^. Both *H* and *A* loci are about 200 kb in length and constitute the supergene, which includes three genes, *dsx, UXT* and *Nach-like*, each of which has dimorphic structure and mimetic and non-mimetic types. In addition, dimorphic genes in the mimicry supergene show similar expression profiles in the pupal wings of both *Papilio* butterflies. However, the wing colouration patterns for non-mimetic and mimetic females of the two closely related butterfly species are not similar. This suggests that different gene networks are driven by mimetic Dsx between the two species. Although the detailed mechanism underlying different wing colouration patterning is not understood fully, further molecular analyses will reveal the evolutionary scenario necessary to produce the diversified wing colouration patterns between *P. polytes* and *P. memnon*.

## Methods

### Insect rearing

Adult *P. polytes* were purchased from Chokan-kabira (Okinawa, Japan) and, for experimentation, those which hatched from the eggs laid by the purchased adults were used. The larvae were fed the leaves of *Citrus natsudaidai* (Rutaceae) or on an artificial diet, and were kept at 25 °C under long-day conditions (16-h light period, 8-h dark period). Adults were supplied with commercial sports drinks and were allowed to breed at 15 °C.

### Quantitative real-time-polymerase chain reaction

The excised sample was washed with phosphate-buffered saline (PBS), transferred to a 1.5 ml sample tube and homogenised in 500 μl TRI reagent (Sigma). Next, 80 μl chloroform was added and mixed well using a vortex-mixer, followed by centrifugation at 15,000 rpm at 4 °C for 10 min. After collecting the supernatant in a fresh tube, an equal volume of isopropanol was added to the supernatant and mixed well, and the mixture was allowed to stand at room temperature for 10 min. Then, the sample was centrifuged at 15,000 rpm at 4 °C for 10 min. After decanting away the supernatant, 500 μl (v/v) 80% ethanol was added to the pellet that was resuspended and centrifuged at 15,000 rpm at 4 °C for 5 min. After discarding the supernatant, the washed pellet was dissolved in 5 μl nuclease-free water. The concentration of RNA dissolved in the nuclease-free water was measured using a spectrophotometer (NanoDrop D-1000) and the absence of degradation of the RNA was confirmed by agarose gel electrophoresis using 2 × formamide Dye. DNase I treatment was performed on the extracted RNA to degrade DNA. In DNase I treatment, 1.5 μl DNase I reaction buffer (20 mM Tris–HCl (pH 8.4), 2 mM MgCl_2_, 50 mM KCl), 2.6 μl nuclease-free water, 0.4 μl RNase Inhibitor, 0.5 μl DNase I (5 U/μl), was added to 2 μl 100 ng/μl RNA and reacted for 15 min at 37 °C. An aliquot (1 μl) 10 mM EDTA was added to the reaction mixture that was inactivated by incubation for 10 min at 65 °C. cDNA synthesis was carried out using Verso cDNA synthesis kit. Reverse transcription was carried out at 42 °C for 30 min, then inactivated by incubation at 95 °C for 2 min, then diluted with double-distilled water to a final concentration of 500 ng/μl. cDNA was prepared from the sample tissue and used as a template according to the method described above. The StepOne™ Real-Time PCR System (ABI) was used to carry out quantitative real-time qPCR. Analysis was carried out using StepOne™ Software v 2.1 by the relative standard curve method. PCR reaction was carried out using Power SYBR Green PCR Master Mix at 95 °C for 15 s and 60 °C for 1 min for 40 cycles. Supplementary Data 1 summarises the primers used for quantitative PCR.

### Functional analysis by RNAi in hindwing using in vivo electroporation method

The siRNA to be injected was designed using siRNA design support software siDirect version 2.0 (http://sidirect2.rnai.jp/)^45,46^. After obtaining the sequence of the open-reading frame region of the target gene from PapilioBase, candidate sequences were searched for, using siDirect and based on the sequence information. To reduce the off-target effect, we used the BLAST search function of PapilioBase to investigate the specificity of the designed sequence to the target gene, and selected one with high specificity from the candidate sequences. Synthesis of siRNA was contracted to FASMAC Corporation. Supplementary Data 1 summarises details of the prepared siRNAs. The siRNA was dissolved in annealing buffer (100 mM CH_3_COOK, 2 mM Mg (CH_3_COO)_2_, 30 mM HEPES–KOH, pH 7.4) to 500 μM, which was diluted further to 250 μM for injection. For the injection, the microinjector FemtoJet was used, and the glass needle for injection was fabricated by using a puller to process a glass tube with a core. The siRNA was drawn up into the glass needle and placed in a micromanipulator M 401 with a glass needle set under a stereoscopic microscope and 1 μl was injected along the wing vein in the hindwing of the pupa. After injection, siRNA was introduced (five square pulses of 7.5 V, 280 ms width) by electrostimulation using an electroporator^19^. At that time, 1% PBS gel and a PBS water drop were placed between the back wing and the electrode as a buffer solution. The detailed method follows that described in the previous paper^47^. The pictures of all the individuals who performed the function analysis are described collectively as Supplementary figures.

### Screening of DE genes in response to *dsx-H* RNAi

As an experimental method, the following analysis procedure was carried out. First, using the in vivo electroporation method, knock-down analysis was performed by introducing siRNA targeting *dsx-H* to the hindwing of the *P. polytes* in a just-pupated (P0) individual. At this time, siRNA was negatively ionised in the aqueous solution, so it was considered to be attracted to the positive electrode side. Therefore, siRNA was considered to be mosaic-like in the PBS region charged to the plus-wing-plus. Then, using a digital camera AxiCam (Carl Zeiss), we photographed the PBS-drop area on the hindwing and grasped the si-*dsx-H*-treated area. On the first day to the third day (P1–3: wing compartment, an area bounded by wing veins, becomes more distinguished and the peripheral region outside of the bordering lacuna gradually degenerates with time) immediately after pupation, the PBS-drop region in the hindwing was sampled based on the photographed image, and the RNA was extracted and purified by the above method. From the position of the wing vein, since the same position can also be specified in the si-*dsx-H*-untreated wing, the PBS-drop region was similarly sampled in the si-*dsx-H*-untreated wings, and RNA was extracted and purified by the above method. Next, by preparing cDNA from the purified RNA and performing quantitative PCR, a sample whose *dsx-H* expression level decreased in the si-*dsx-H*-treated wing, compared to an untranslated wing with si-*dsx-H*, was selected, and was used as a sample for RNA-Seq analysis (each individual, *n* = 1, in P1–3). BGI Japan Corporation was contracted to carry out the RNA-Seq analysis. Sequencing was carried out with 100 bp paired-end reads using the Illumina HiSeq 2500 platform. The obtained read data was mapped to the Ppolytes.v1.0.0. transcriptome^17^ using the analysis software Bowtie 2^20^. Genes whose expression varied under the control of *dsx-H* were identified by comparing gene expression levels from the mapping status, using analysis software DESeq^21^. After calculating the expression level of each gene as the FPKM (Fragments Per Kilobase of transcript per Million mapped reads) value, genes with significantly different expression levels (*P* < 0.05) in comparisons between si-*dsx-H* untreated wing and si-*dsx-H* treated wing were selected as genes showing differential expression. Figure 2 and Supplementary Fig. 2 show the genes whose expression levels were reduced or increased by introducing si-*dsx-H*, respectively (DESeq, *P* < 0.05). Both gene lists consist of TFs and signalling molecules based on Gene Ontology terms ‘transcription’, ‘signalling receptor binding’ or ‘signalling receptor activity’. Heatmaps were generated in R Bioconductor using the heatmap.2 function of the gplots package (http://cran.r-project.org/web/packages/gplots/index.html).

### Construction of phylogenetic trees

Wnt1/6 amino acid sequences were obtained from the following database for each species: *B. mori* (Wnt1:NP_001037315.1, Wnt6:XP_012548361.1) (KAIKObase: http://sgp.dna.affrc.go.jp/KAIKObase/), *Danaus plexippus* (Wnt1:EHJ69660.1, Wnt6:EHJ69658.1) (Monarchbase: http://monarchbase.umassmed.edu), *P. polytes* (Wnt1:PpolytesGene0008023, Wnt6:PpolytesGene0008021) and *Papilio xuthus* (Wnt1:PxuthusGene0002332, Wnt6:PxuthusGene0002330) (PapilioBase: http://papilio.bio.titech.ac.jp/), *Papilio machaon* (Wnt1: KPJ11870.1, Wnt6: XP_014362722.1) (RefSeq database: http://lepbase.org). *P. memnon Wnt1/6* nucleotide sequences were obtained from a previous study of the *P. memnon* genome^37^. Alignment was conducted using ClustalW programme implemented in MEGA5^48^, and employing the GTR + G model (a gamma model for rate heterogeneity) maximum-likelihood method. Reliability of the systematic relationship was judged by the bootstrap method (1000 times repeats).

### Quantification of pattern area in hindwing using Image J

After emergence of the lab-bred female (*HH*: *n* = 22; *Hh*: *n* = 41; *hh*: *n* = 32), the lab-bred male (*HH*: *n* = 8; *Hh*: *n* = 21; *hh*: *n* = 37), their hindwings were excised from the base of the wing and photographed the entire wing for each individual using a digital microscope VH-5500SP1344 (Keyence). The image was captured using the analysis software Image J^49^ and the proportion of both the pattern area (red, white, black) of the *P. polytes* female posterior wing relative to the total area of the hindwing and the pattern area of the non-mimetic female and male hindwing relative to the total area of the hindwing (White/black colour ratio) was calculated. First, we recognised only the colour (prescribed red this time) in the wing print from the brightness of the picture of Image J and the area of the recognised area was calculated. Next, by converting the photo to black and white (8-bit image), only the white and the black regions (the red region was converted to the black region) were converted, and the areas of the white region and the black region were calculated. From the calculated area, the value obtained by dividing the area of the red region or the white region by the area of the whole wing was regarded as the ratio of the red area (or white area) in the hindwing.

### Statistics and reproducibility

Statistical analysis was performed at least in three biological replicates, except for RNA-seq. All data are presented as mean ± standard deviation (SD). All statistical tests were two-sided unless indicated otherwise. Statistical differences were analysed using Student’s *t*-test; *p* < 0.05 was considered statistically significant.

### Reporting summary

Further information on research design is available in the Nature Research Reporting Summary linked to this article.

## Supplementary information

Supplementary Information

Description of additional supplementary items

Reporting Summary

Supplementary Data 1

Supplementary Data 2

Supplementary Data 3

Supplementary Data 4

## Data Availability

All raw sequencing data has been deposited in the DNA data bank of Japan. Accession information: short-read archive for the *P. polytes* RNA sequences accession ID, DRR140179–DRR140184. No restrictions apply to access of all the data. The individual data points plotted in the main figures are reported in Supplementary Data 2.
