# Supplementary material for: The mimetic wing pattern of Papilio polytes butterflies is regulated by a doublesex-orchestrated gene network
Source: Commun Biol. 2019 Jul 10;2:257. doi: 10.1038/s42003-019-0510-7 (PMC6620351; doi:10.1038/s42003-019-0510-7)
Supplement: Supplementary file 1 — Supplementary Information [file 42003_2019_510_MOESM1_ESM.pdf]

Supplementary Figures

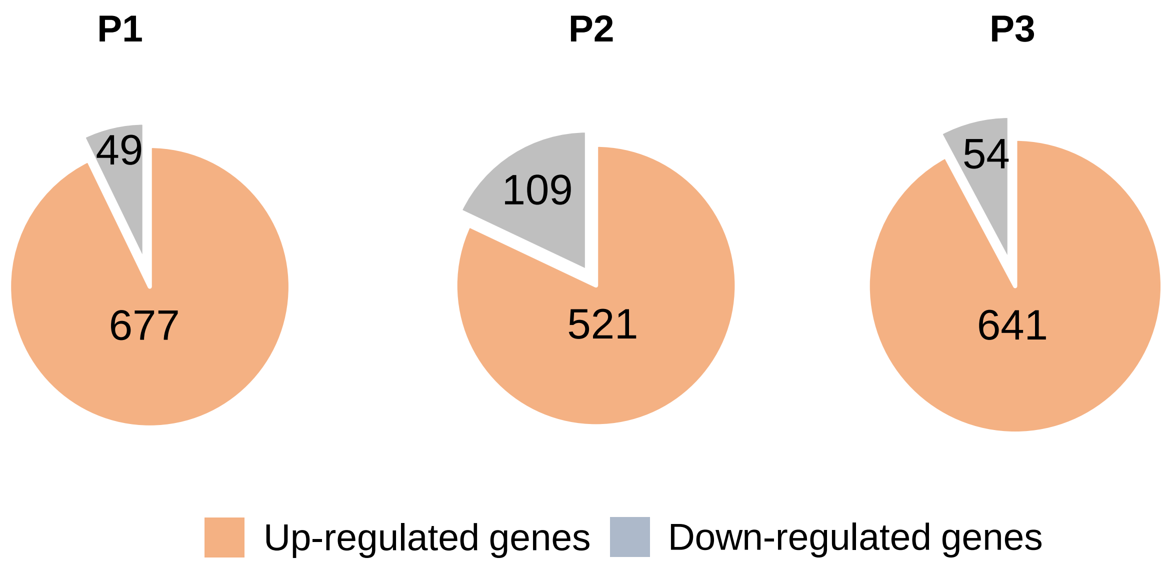

Figure S1 | Ratio of DE genes in response to *dsx-H* RNAi in mimetic hindwing. Each pie chart depicts the number of up-regulated and down-regulated DE genes ($P < 0.05$) during P1 to P3, respectively.

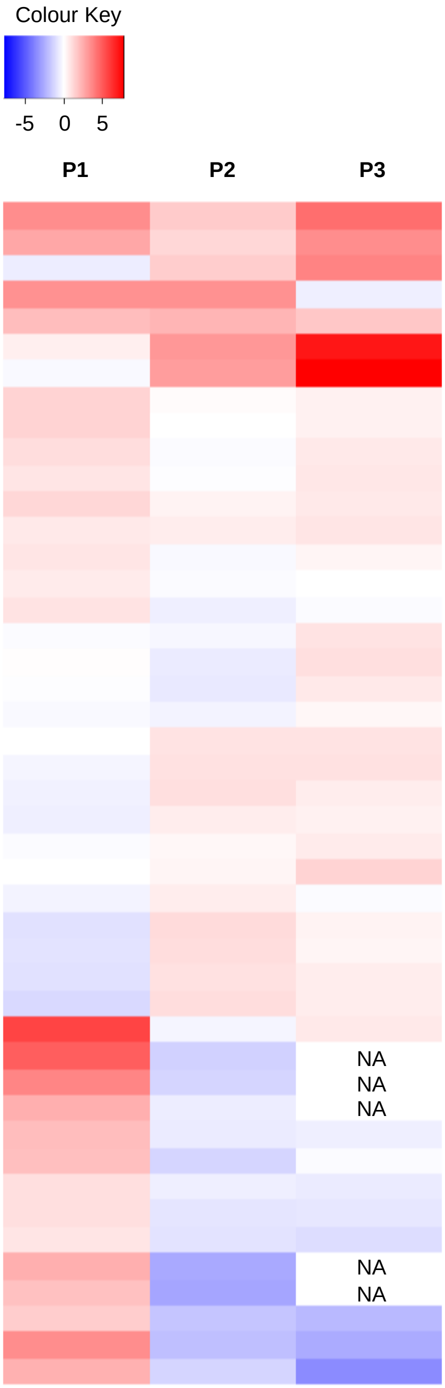

Gene name	Gene ID (PpolytesGene)	Gene function: TF	Gene function: Signaling ligand/receptor	DESeq P1	DESeq P2	DESeq P3
torso-like	0002825		■	✓		✓
Rhodopsin 7	0004305		■	✓	✓	✓
Ets21C	0001455	■			✓	✓
Suppressor of lurcher protein 1	0001905		■	✓		
Fibroblast growth factor receptor 3	0000781		■	✓		
PGRP-LB-like	0009827		■	✓	✓	✓
PGRP-LB-like	0009826		■		✓	✓
Atrial natriuretic peptide receptor 1	0003935		■	✓		
5-Hydroxytryptamine receptor 2B	0009079		■	✓		✓
germ cell expressed	0003689	■		✓		
Tyrosine kinase receptor Cad96Ca	0007443		■	✓		
ZAP1-like	0000571	■		✓		✓
clockwork orange	0007425	■		✓		
numb	0000204		■	✓		
cryptochrome	0000890		■	✓		
Sox14	0005714	■		✓		
knirps-like	0007352	■				✓
broad	0011858	■				✓
torso-like	0002826		■			✓
vrille	0008803	■				✓
rhea	0008748		■		✓	✓
E74	0006891	■			✓	✓
methuselah-like 10	0012011		■		✓	✓
Hormone receptor 3	0005922	■			✓	
Macrophage mannose receptor 1	0011415		■			✓
Mitf	0008870	■				✓
similar	0004200	■			✓	
Myospheroid	0008017		■		✓	✓
moody	0006873		■		✓	
Insulin-like receptor	0011792		■		✓	✓
Fer3	0004208	■			✓	
Pyrokinin 1 receptor	0001605		■	✓		
Allatostatin C	0012169		■	✓		
zinc finger protein 2	0008978	■		✓		
hamlet-like	0009332	■		✓		✓
hamlet	0009331	■		✓		
Grip	0007407		■	✓		
CREB3 Regulatory Factor	0004931	■		✓		
apolipophorin	0008747		■	✓		
cut	0008570	Homeobox		✓		✓
optomotor-blind-related-gene-1	0009648	■		✓		
Grik	0004952		■	✓		
Mohawk	0007758	Homeobox		✓		
abdominal-A	0004024	Homeobox		✓		
Neuroligin 1	0009146		■	✓		

Figure S2 | Up-regulated DE genes in response to *dsx-H* knockdown. Heat map of DE genes up-regulated in a mimetic female in RNA-seq with *dsx-H* knockdown. The colour key indicates scaled log-two-fold change value (Log2-fold change value = Log2 siRNA-treated FPKM–Log2 untreated FPKM). Bars coloured orange and blue in the Gene function column represent transcription factor and signalling molecules, respectively. Check marks in the DESeq column indicate $P < 0.05$ in the DESeq analysis. NA, not applicable (= missing value).

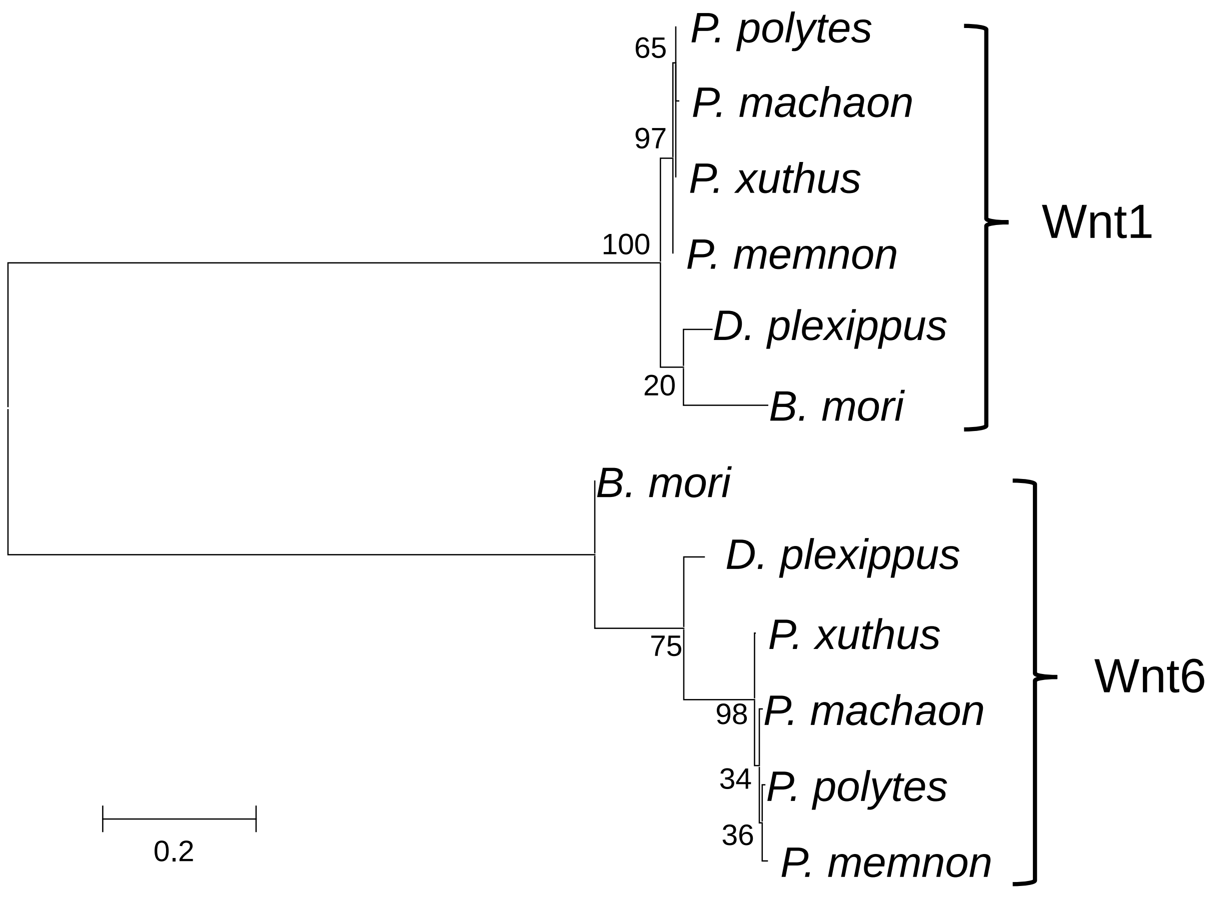

Figure S3 | Phylogenetic trees of Wnt1 and Wnt6 proteins. The phylogenetic tree of Wnt1 and Wnt6 proteins (maximum-likelihood method using GTR+G model). A total of 310 Wnt1 sites and 353 Wnt6 sites were used. Bootstrap values are shown on the branches.

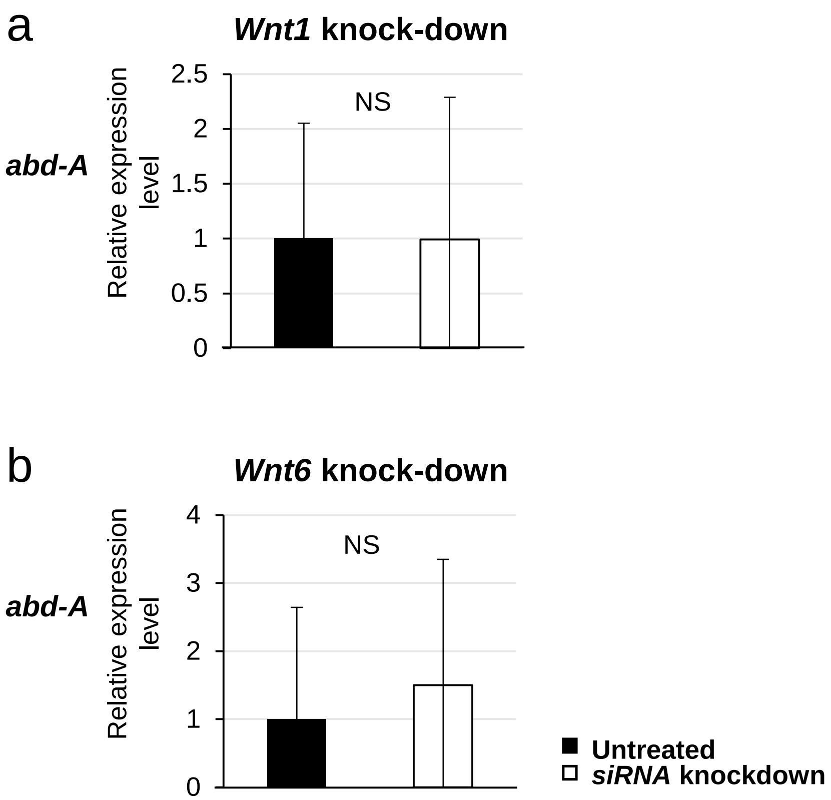

Figure S4 | Expression levels of *abd-A* in *Wnt1/6* knock-down hindwings of mimetic female.

(a, b) Relative expression level of *abd-A* in *Wnt1* (a) and *Wnt6* (b) knock-down hindwings of mimetic female. The expression levels are shown as relative values with the expression level in the untreated wing of mimetic female (*Hh*) taken as 1. We estimated the gene expression levels by real-time RT-PCR using RpL3 as an internal control. Error bars show standard deviation (a, n = 7; b, n = 5). Student's t test (NS, not significant).

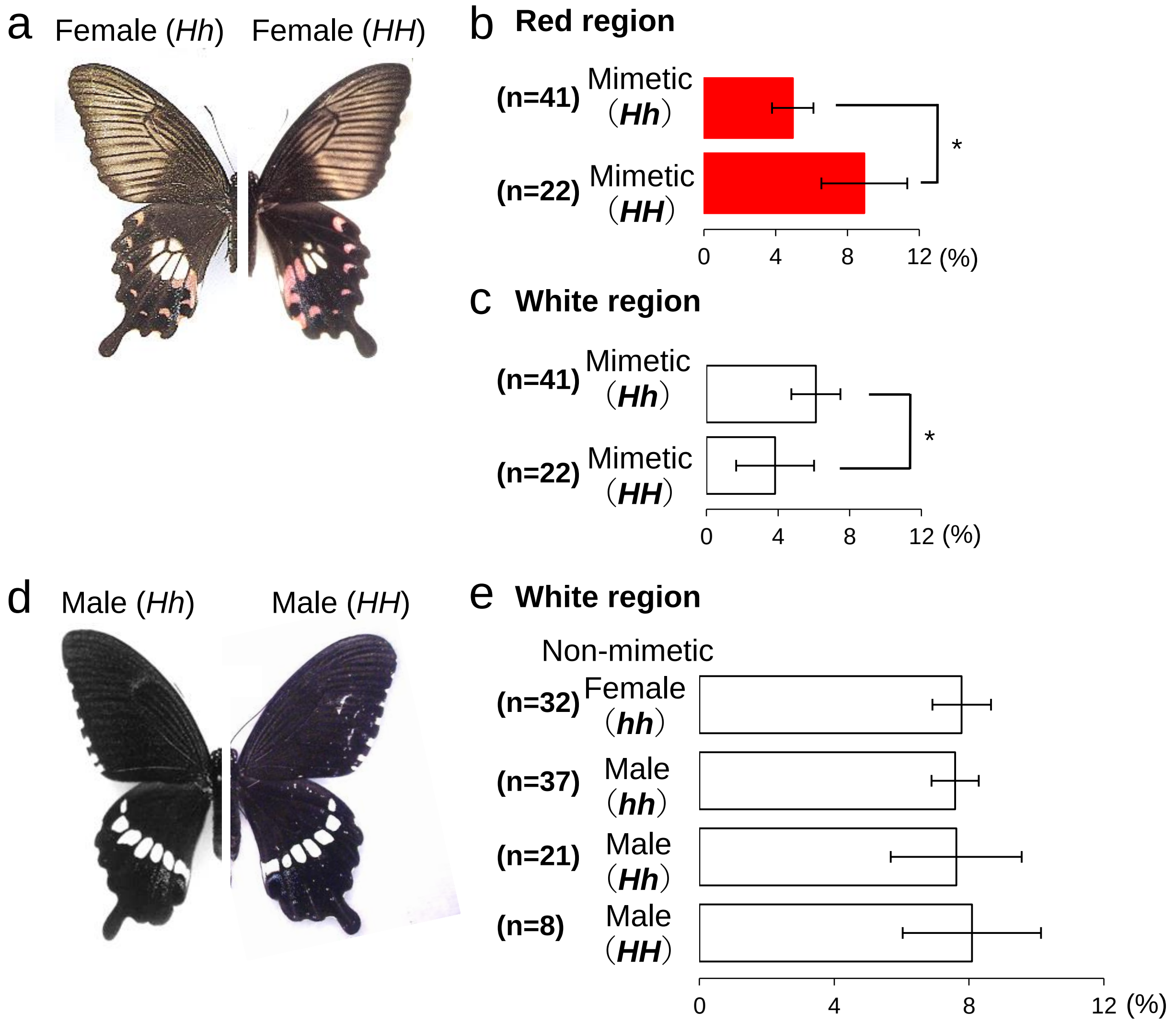

Figure S5 | Percentages of red and white regions to the entire hindwings. (a) Wing patterns of mimetic female in H heterozygote (*Hh*) and homozygote (*HH*). (b, c) Percentages of red (b) and white region (c) to the entire mimetic hindwings (*Hh* and *HH*). (d) Wing patterns of male in *Hh* and *HH*. (e) Percentage of white region in non-mimetic female (*hh*) and male (*hh*, *Hh* and *HH*) to the entire hindwings. Error bars show standard deviation of 3 experimental replicates. *: $P < 0.05$ for Student's *t* test.

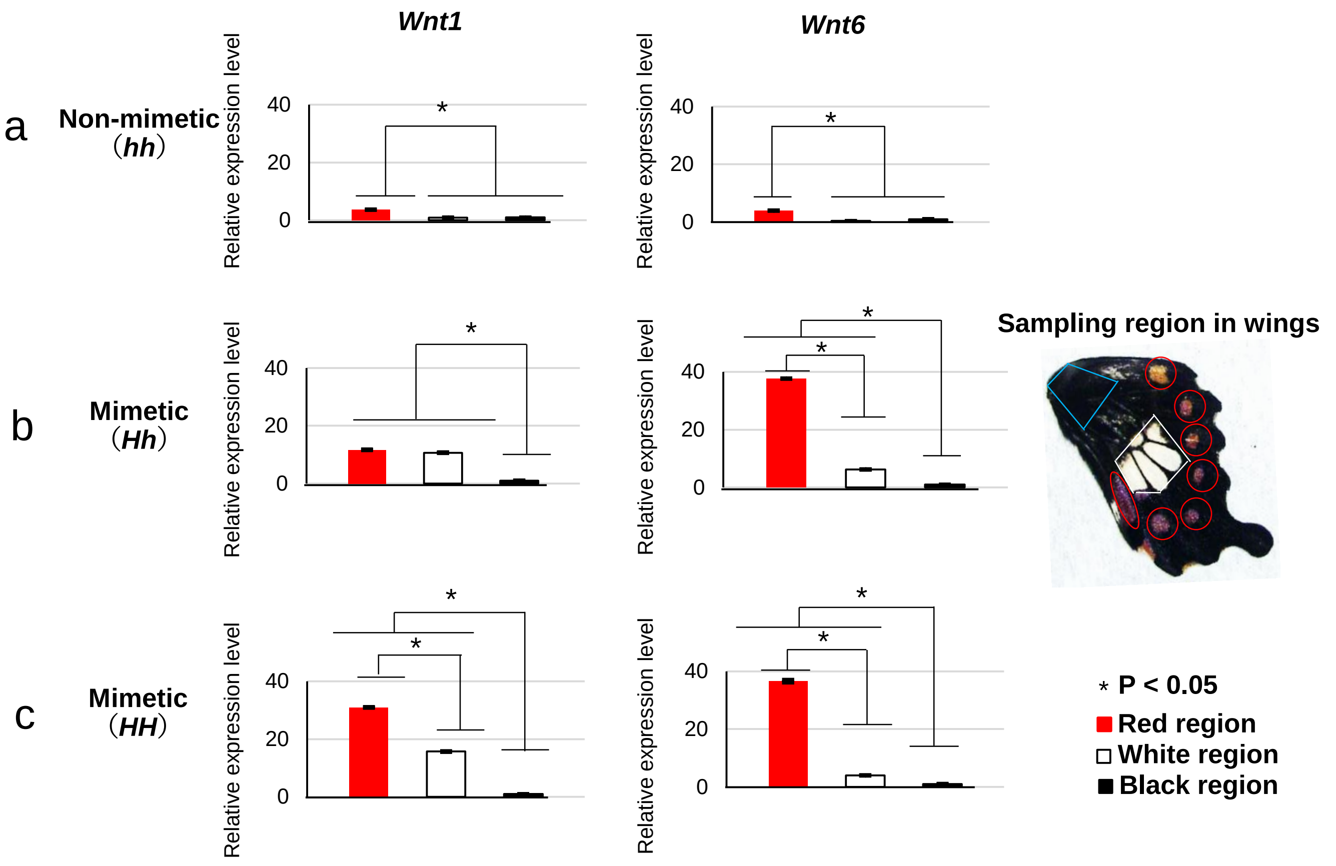

Figure S6 | Spatial expression pattern of *Wnt1* and *Wnt6* in female hindwings. The expression levels of *Wnt1* and *Wnt6* in (a) non-mimetic female (*hh*), (b, c) mimetic female (*Hh*, *HH*) in P2 period. The expression levels are shown as relative values, with the expression level of the black region set to 1. Bars colored red, white and black represent samples from red, white and black regions (or corresponding regions) of mimetic and non-mimetic pupal hindwings, respectively (schematic at right). We estimated the gene expression levels by real-time RT-PCR using *RpL3* as an internal control. Error bars show standard deviation of 3 experimental replicates. *: *P* <0.05 for Student's t test.

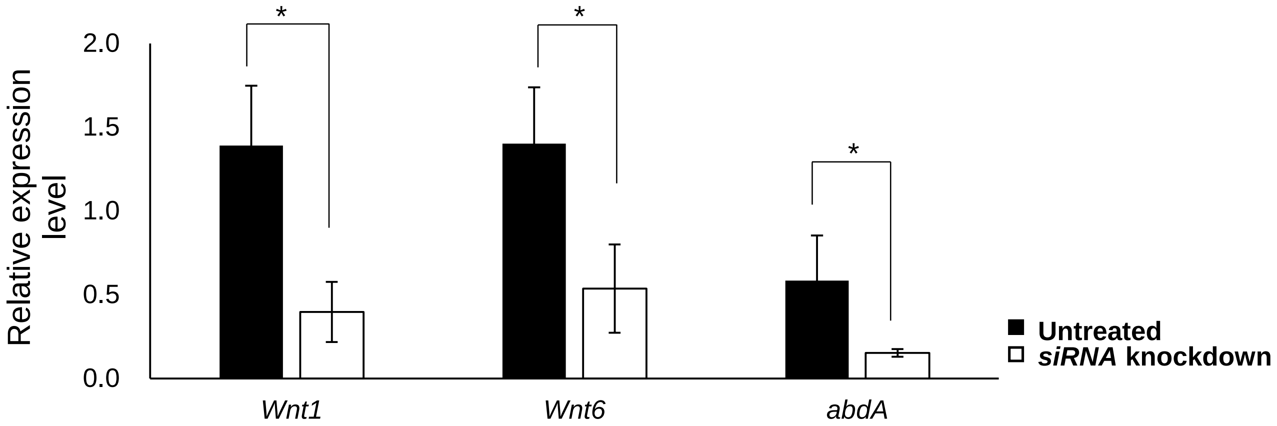

Figure S7 | Verification of the reduction of target genes in RNAi experiments. Expression levels of *Wnt1*and *Wnt6* in mimetic female (*Hh*) and *abd-A* in non-mimetic female (*hh*) are shown. We estimated the ratio of the target gene reduction between untreated (black bar) and treated wings (white bar) by each siRNA in the same individual by real-time RT-PCR using *RpL3* as an internal control. Error bars show standard deviation of 3 biological replicates. *: $P < 0.05$ for Student's t test.

Wnt1 siRNA (continued on next page)

a

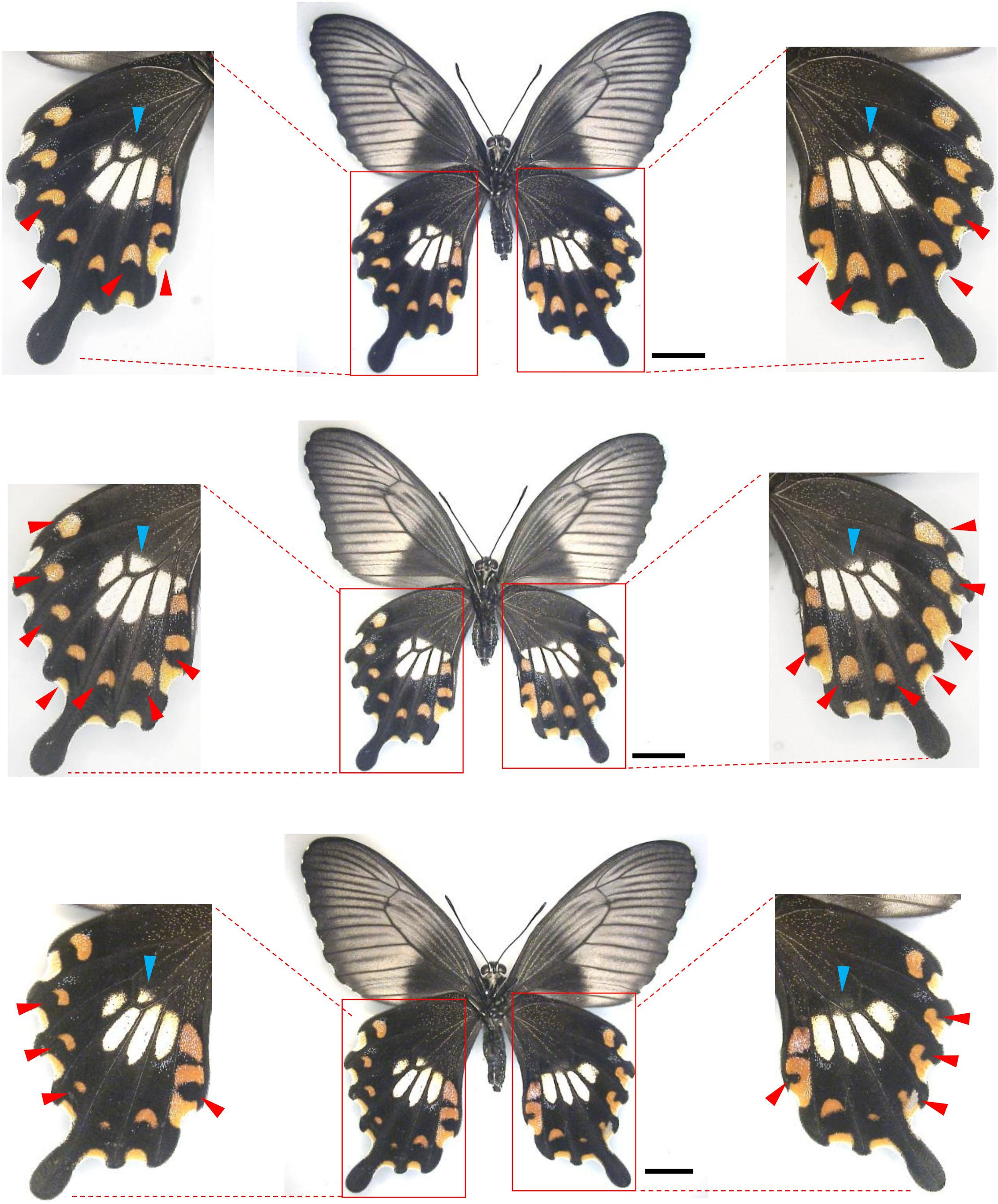

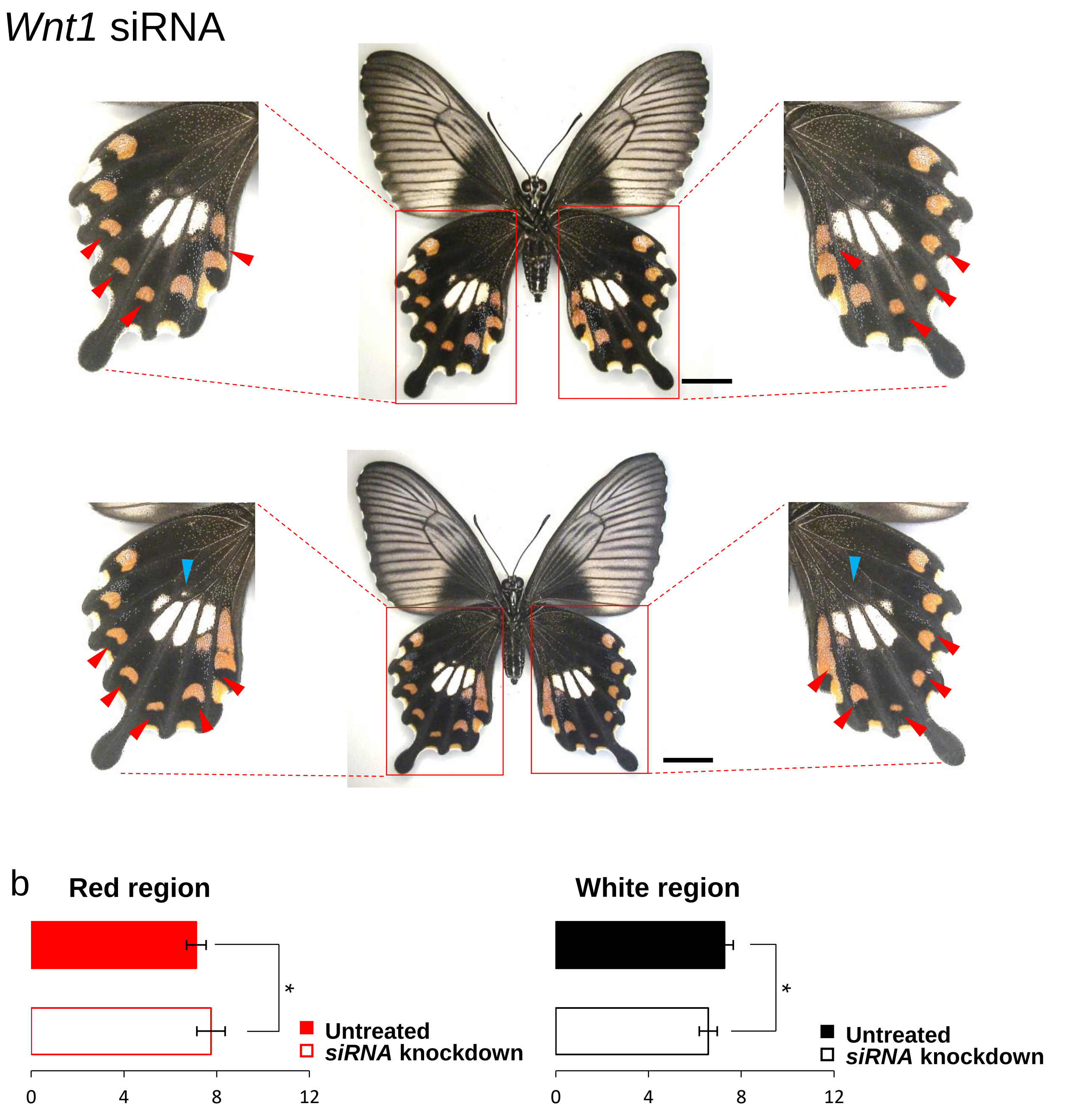

Figure S8 | RNAi phenotypes of *Wnt1* in mimetic hindwings. (a) Five *Wnt1* knockdown phenotypes are shown. siRNA was injected into the pupal hindwing just after pupation and electroporated in the ventral side of hindwing. Red and blue arrowheads represent the changed red and white regions, respectively. Scale bars, 1cm. (b) Percentages of red and white regions to the entire hindwings in siRNA knockdown individuals. Error bars show standard deviation of 6 experimental replicates. *: $P < 0.05$ for Student's t test.

Wnt6 siRNA (continued on next page)

a

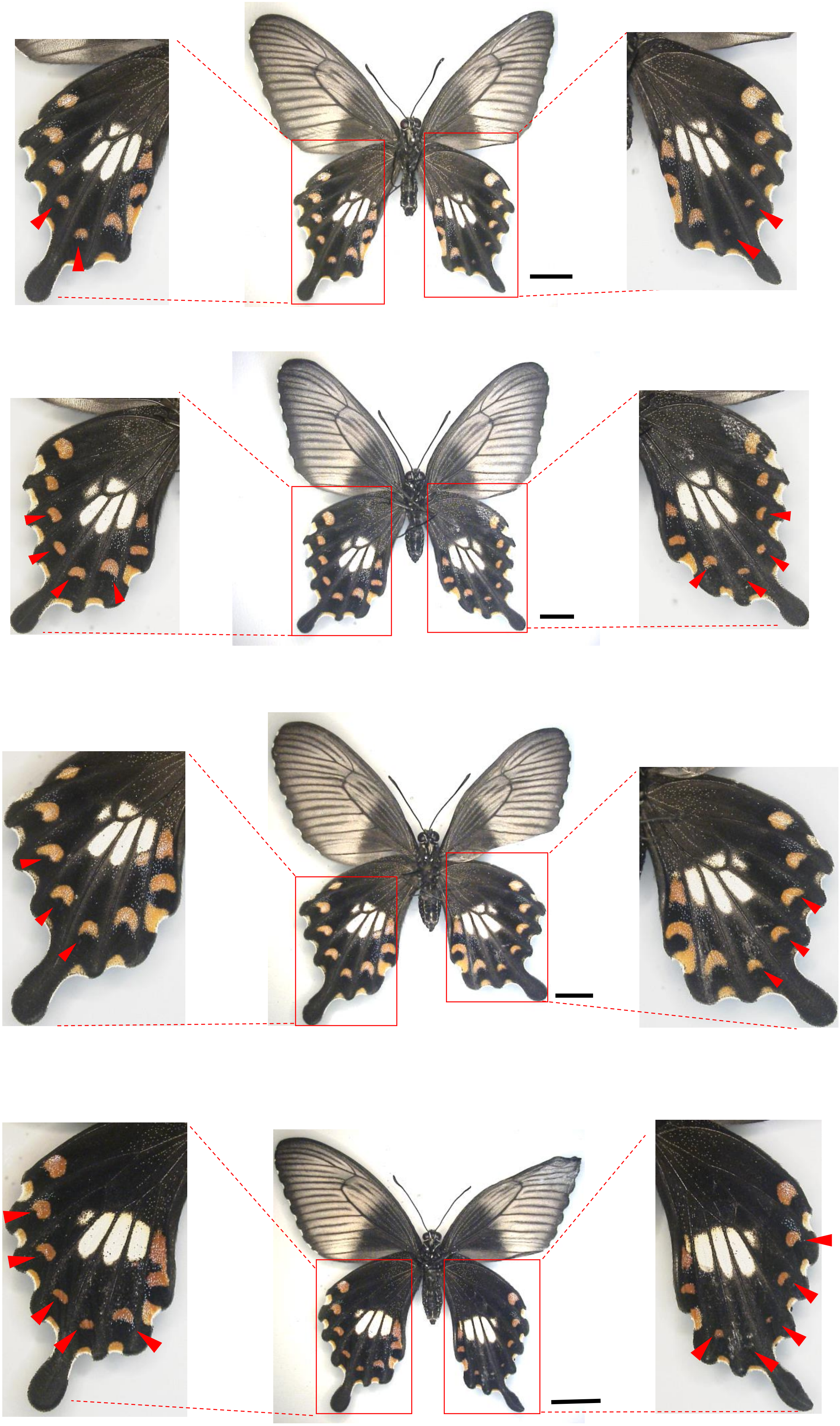

Wnt6 siRNA

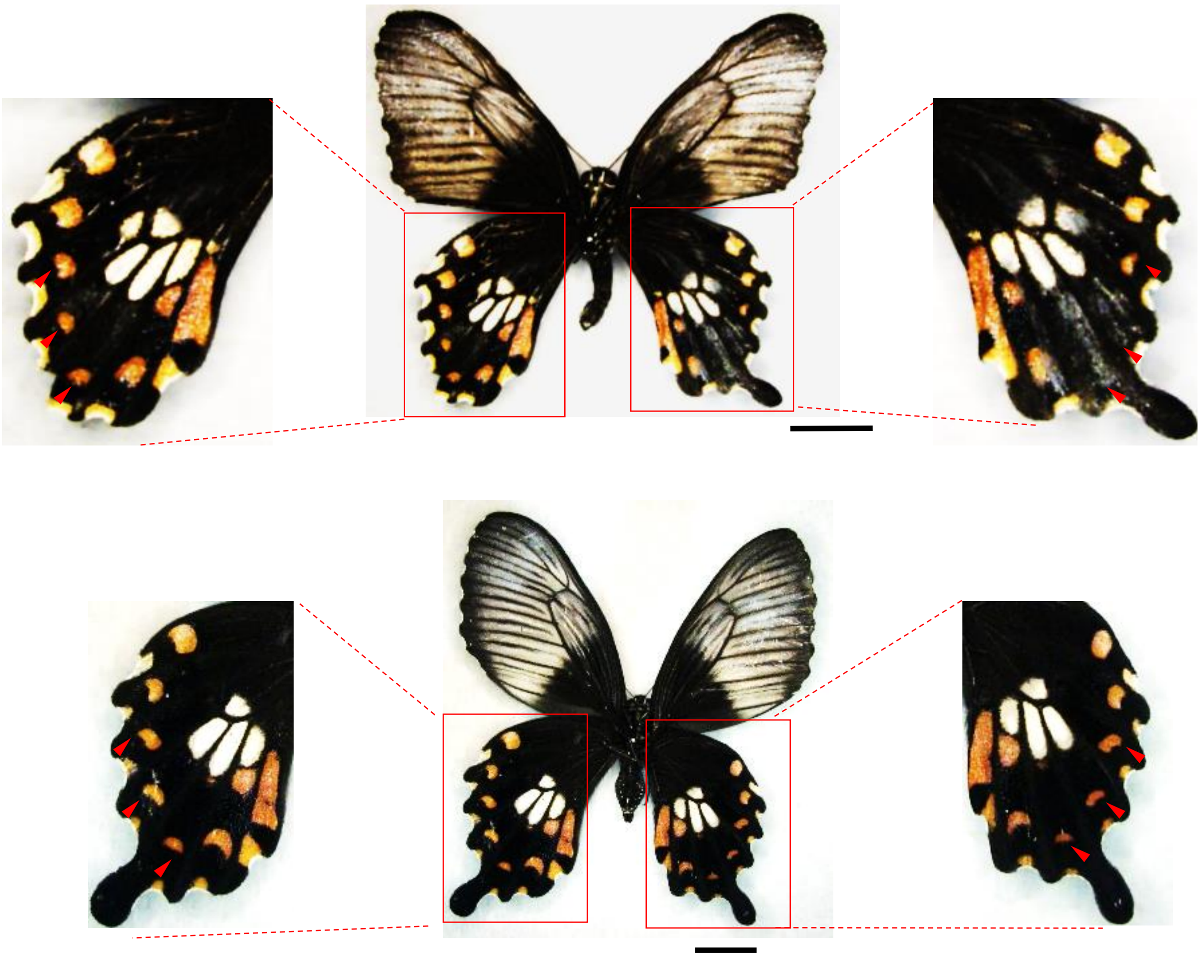

b

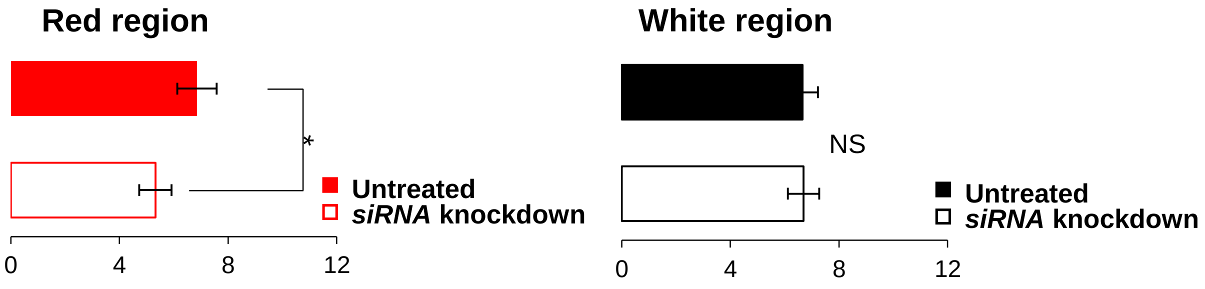

Figure S9 | RNAi phenotypes of *Wnt6* in mimetic hindwings. (a) Six *Wnt6* knockdown phenotypes are shown. siRNA was injected into the pupal hindwing just after pupation and electroporated in the ventral side of hindwing. Red and blue arrowheads represent the repressed red and white regions, respectively. Note that some phenotypes have a few scales with transparent looking due to artificial damage in the process of making butterfly specimens. Scale bars, 1cm. (b) Percentages of red and white regions to the entire hindwings in siRNA knockdown individuals. Error bars show standard deviation of 7 experimental replicates. *: $P < 0.05$ for Student's t test. NS, not significant.

Wnt1 + *Wnt6* siRNA (continued on next page)

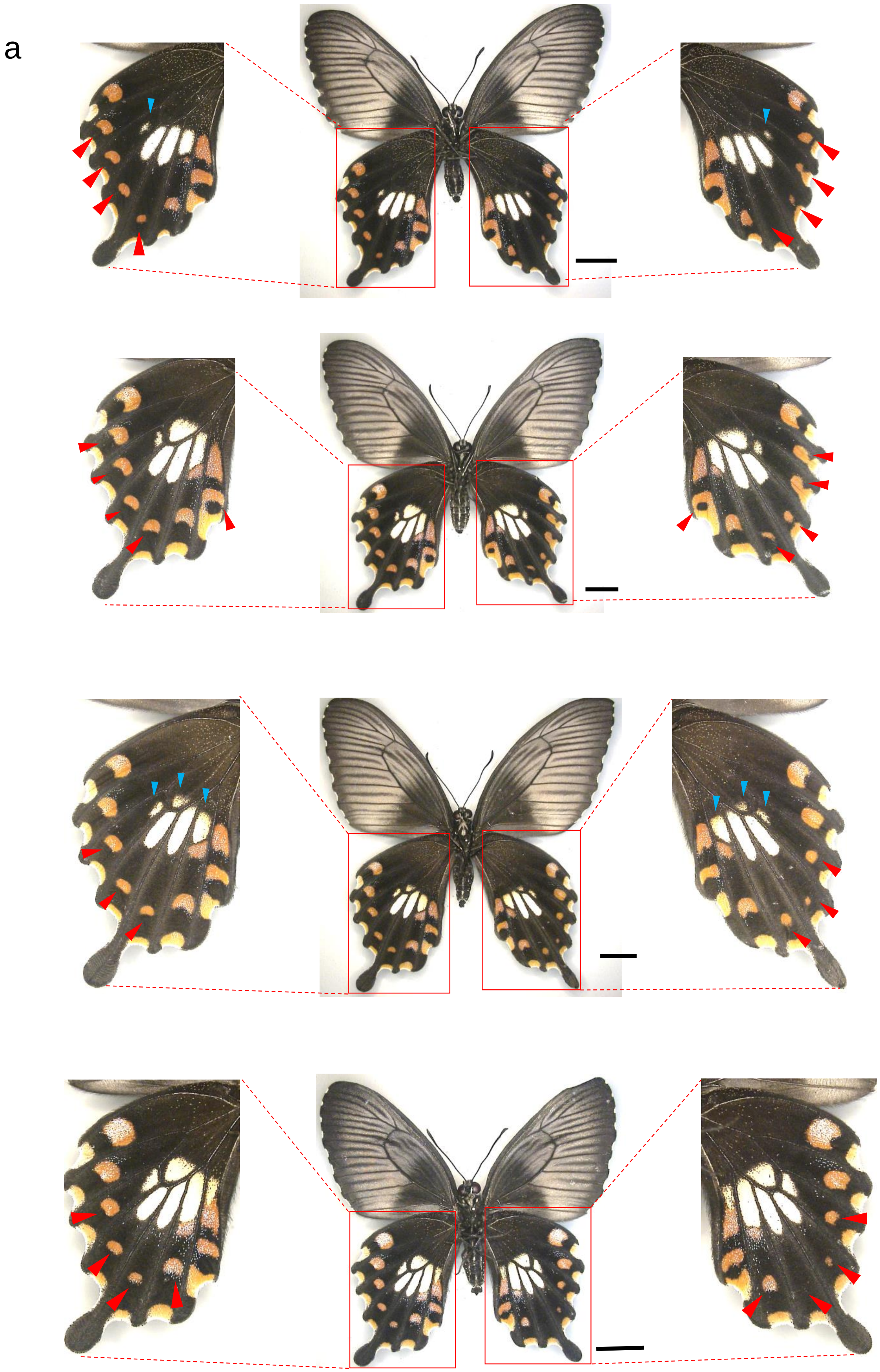

Wnt1 + *Wnt6* siRNA

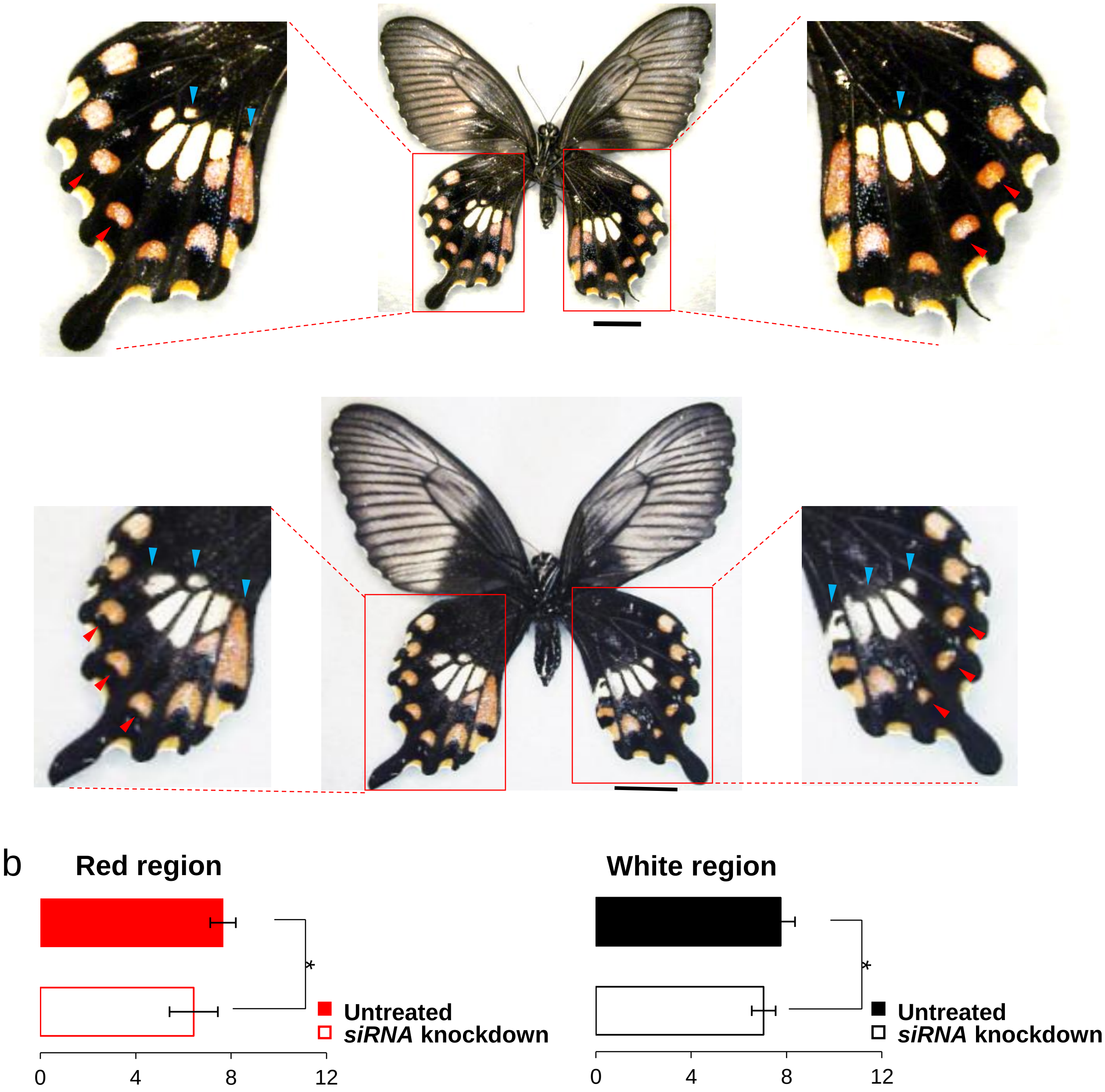

Figure S10 | Double-RNAi phenotypes of *Wnt1* and *Wnt6* in mimetic hindwings. (a) Six *Wnt1* and *Wnt6* double-knockdown phenotypes are shown. siRNA was injected into the pupal hindwing just after pupation and electroporated in the ventral side of hindwing. Red and blue arrowheads represent the repressed red and white regions, respectively. Scale bars, 1cm. (b) Percentages of red and white regions to the entire hindwings in siRNA knockdown individuals. Error bars show standard deviation of 7 experimental replicates. *: P <0.05 for Student's t test.

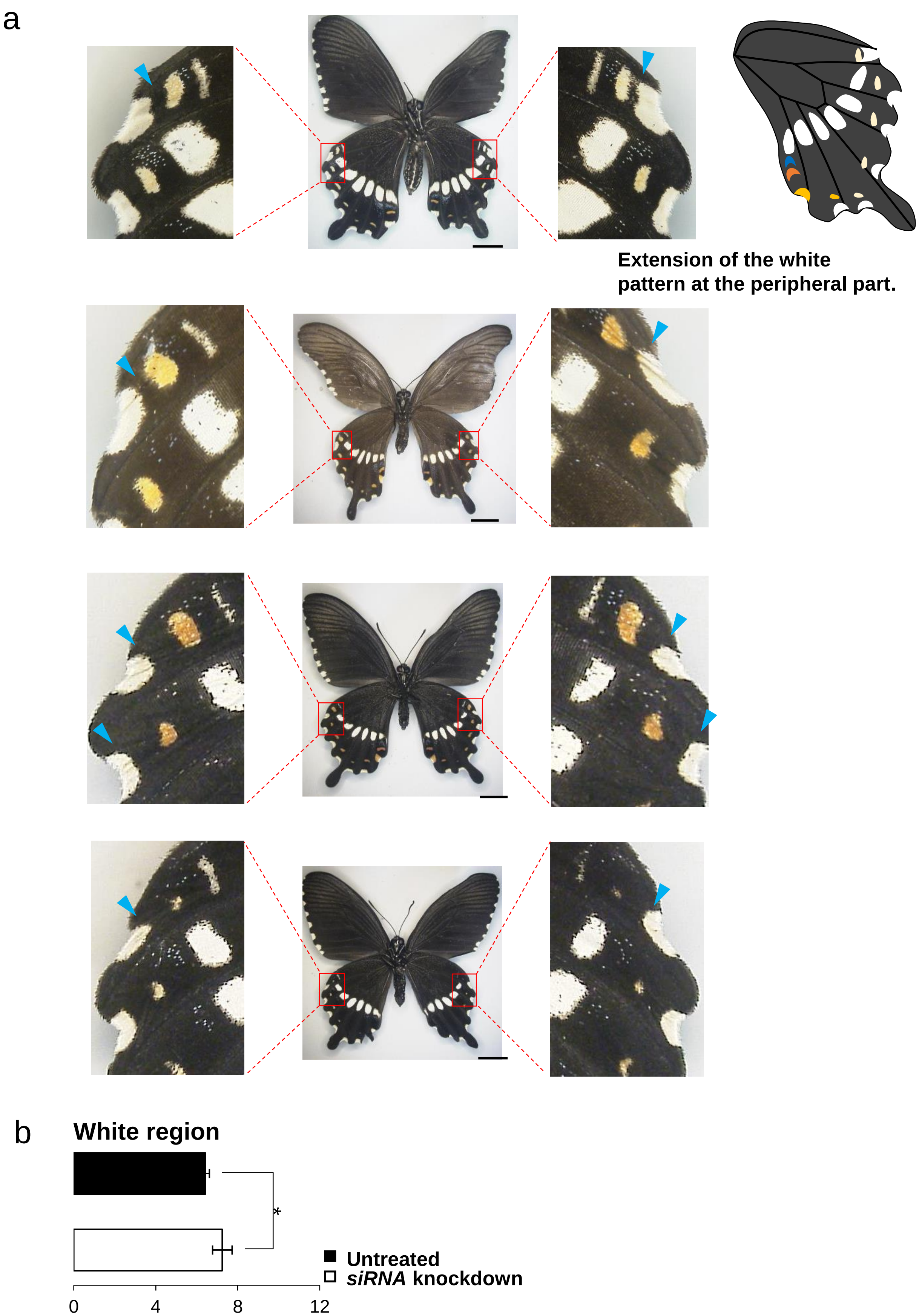

Figure S11 | RNAi phenotypes of *abd-A* in non-mimetic hindwings. (a) Four *abd-A* knockdown phenotypes are shown. siRNA was injected into the pupal hindwing just after pupation and electroporated in the ventral side of hindwing. Blue arrowheads represent the extended white regions (schematic at right). Scale bars, 1cm. (b) Percentages of white regions to the entire hindwings in siRNA knockdown individuals. Error bars show standard deviation of 5 experimental replicates. *: $P < 0.05$ for Student's t test.

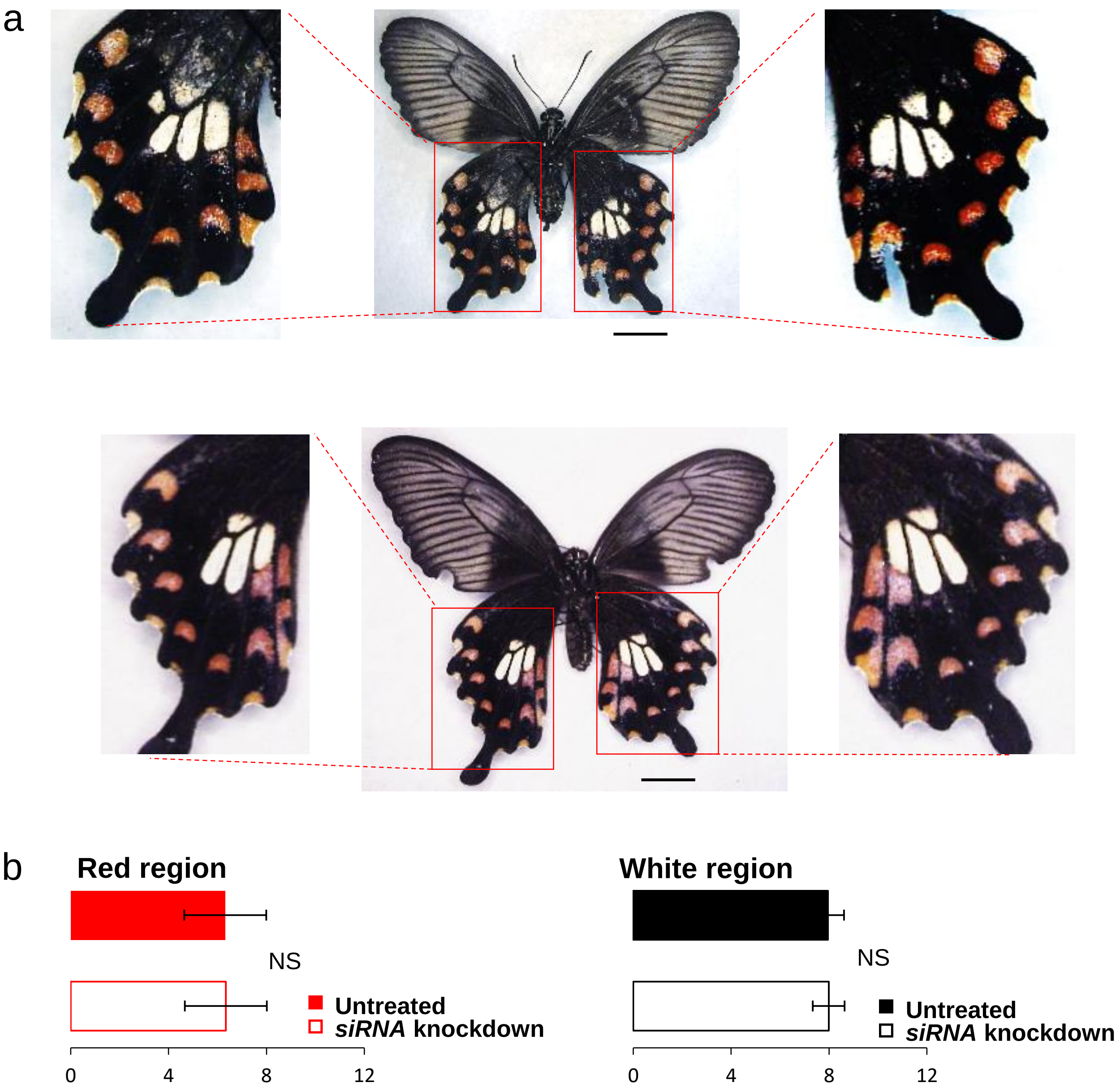

Figure S12 | RNAi effect of *abd-A* in mimetic hindwings. (a) *abd-A* knockdown resulted in no effect in mimetic hindwings (*Hh* heterozygotes). siRNA was injected into the pupal hindwing just after pupation and electroporated in the ventral side of hindwing. Scale bars, 1cm. (b) Percentages of red and white regions to the entire hindwings in siRNA knockdown individuals. Error bars show standard deviation of 3 experimental replicates. Student's t test (NS, not significant).

abd-A siRNA (continued on next page)

a

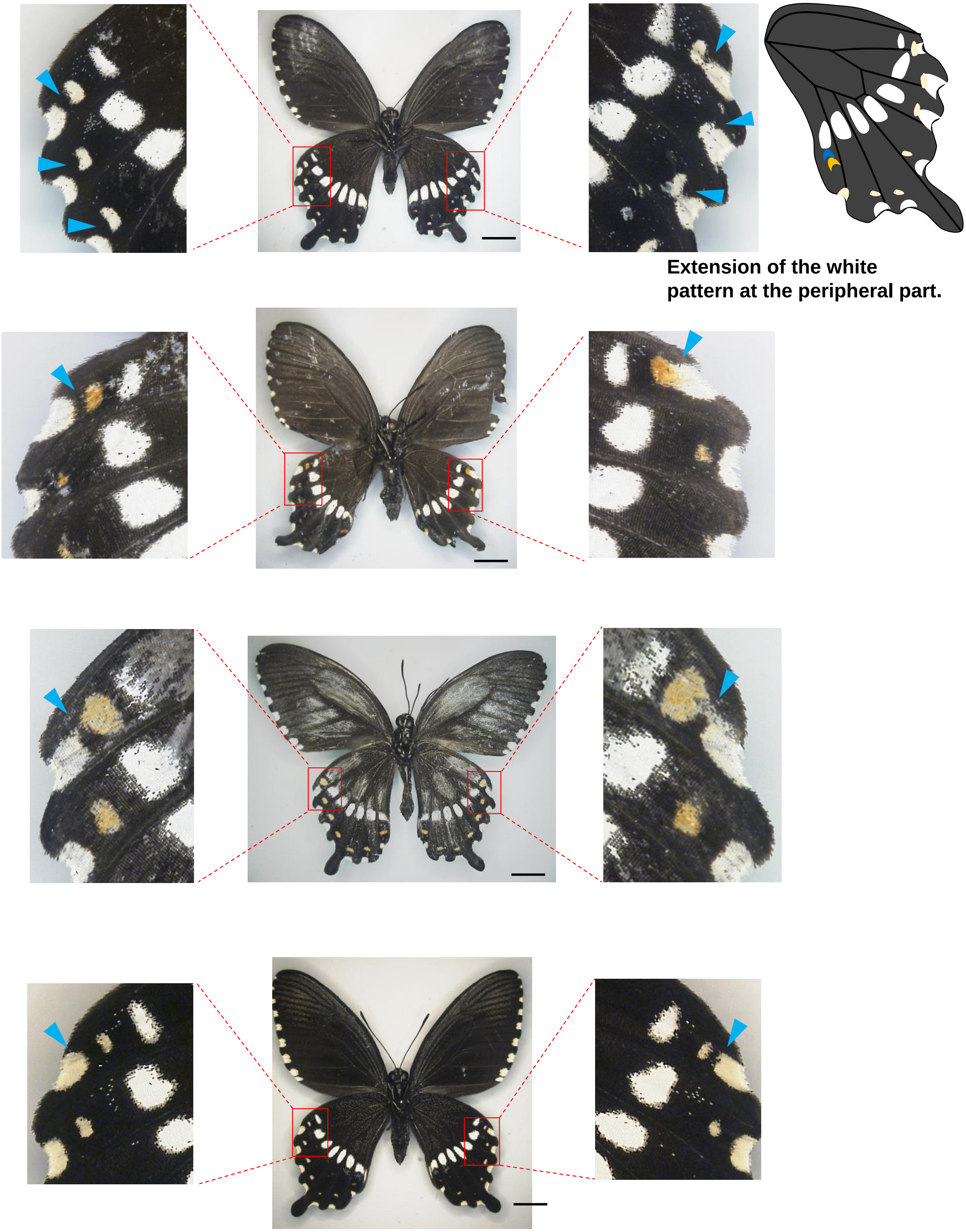

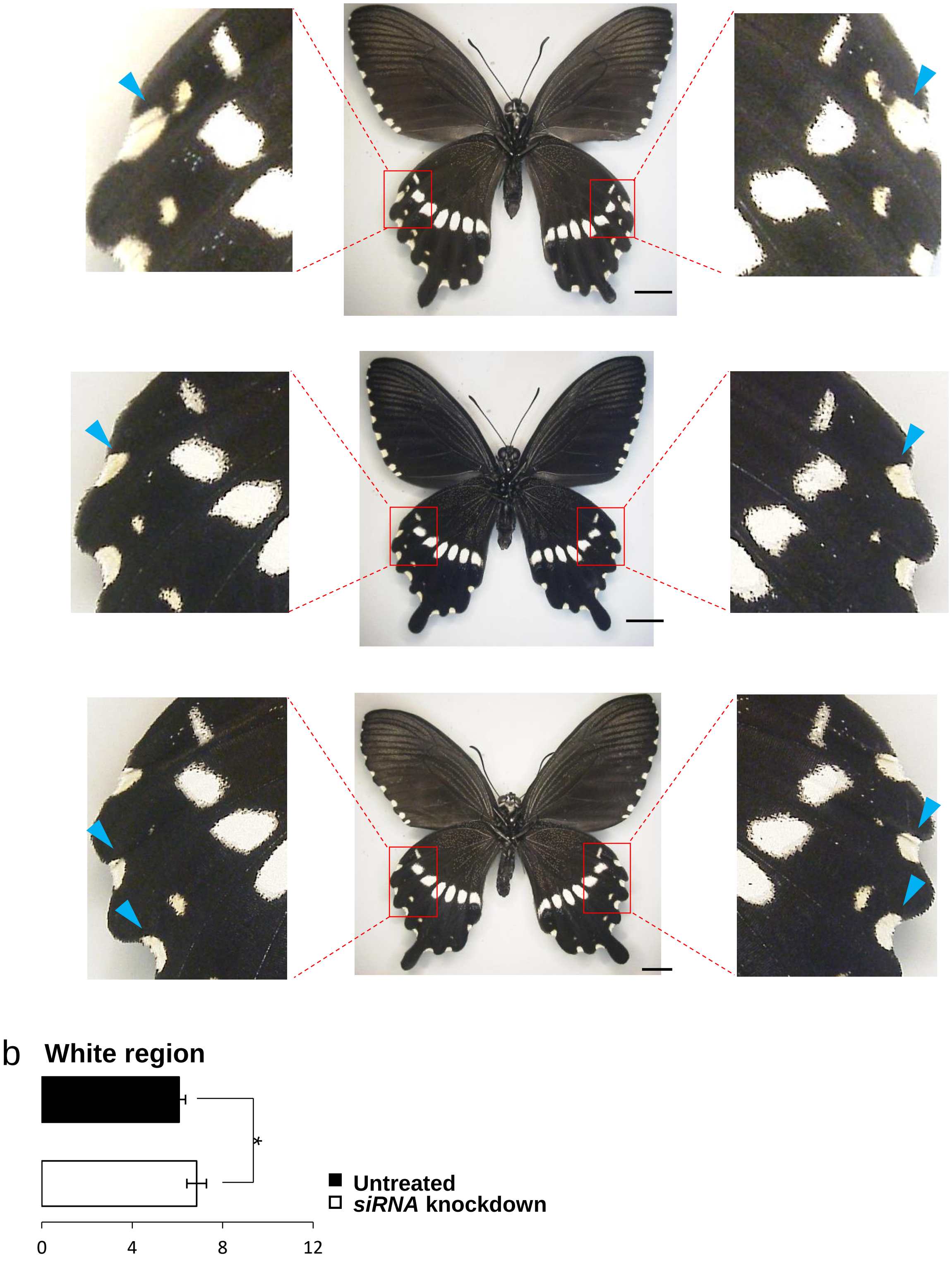

Figure S13 | RNAi phenotypes of *abd-A* in male hindwings. (a) Seven *abd-A* knockdown phenotypes (*Hh* heterozygotes) are shown. siRNA was injected into the pupal hindwing just after pupation and electroporated in the ventral side of hindwing. Blue arrowheads represent the extended white regions (schematic at right). Scale bars, 1cm. (b) Percentages of white regions to the entire hindwings in siRNA knockdown individuals. Error bars show standard deviation of 7 experimental replicates. *: $P < 0.05$ for Student's t test.

Table S1 Summary of RNA-seq data

Sample ID	sex	Phenotype	Genotype	Collectiong tissues	Collecting time	Total size (bp)	Accession ID
P1	Female	Mimetic	*Hh*	Hind wing	P1	28,076,301	DRR140179
P2	Female	Mimetic	*Hh*	Hind wing	P2	35,914,189	DRR140180
P3	Female	Mimetic	*Hh*	Hind wing	P3	25,830,776	DRR140181
P1 siRNA	Female	Mimetic	*Hh*	Hind wing	P1	28,076,301	DRR140182
P2 siRNA	Female	Mimetic	*Hh*	Hind wing	P2	35,914,189	DRR140183
P3 siRNA	Female	Mimetic	*Hh*	Hind wing	P3	25,830,776	DRR140184
