# Supplementary material for: The mimetic wing pattern of Papilio polytes butterflies is regulated by a doublesex-orchestrated gene network
Source: Commun Biol. 2019 Jul 10;2:257. doi: 10.1038/s42003-019-0510-7 (PMC6620351; doi:10.1038/s42003-019-0510-7)
Supplement: Supplementary file 2 — Description of additional supplementary items [file 42003_2019_510_MOESM2_ESM.docx]

**The mimetic wing pattern of *Papilio polytes* butterflies is regulated by *doublesex*-orchestrated gene network**

**Supplementary Data 1. List of qPCR primers and siRNAs.**

**Supplementary Data 2. Source data used to generate the plots and heatmap in main figures.**

**Supplementary Data 3. A separate Excel-editable Table of Fig. 2b**

**Supplementary Data 4. A separate heatmap image in Fig. 2b**
