# Supplementary material for: The mimetic wing pattern of Papilio polytes butterflies is regulated by a doublesex-orchestrated gene network
Source: Commun Biol. 2019 Jul 10;2:257. doi: 10.1038/s42003-019-0510-7 (PMC6620351; doi:10.1038/s42003-019-0510-7)
Supplement: Supplementary file 3 — Reporting Summary [file 42003_2019_510_MOESM3_ESM.pdf]

nature research

Corresponding author(s): Haruhiko Fujiwara

Last updated by author(s): Jun 14, 2019

Reporting Summary

Nature Research wishes to improve the reproducibility of the work that we publish. This form provides structure for consistency and transparency in reporting. For further information on Nature Research policies, see Authors & Referees and the Editorial Policy Checklist.

Statistics

For all statistical analyses, confirm that the following items are present in the figure legend, table legend, main text, or Methods section.

n/a	Confirmed	
☐	☒	The exact sample size (*n*) for each experimental group/condition, given as a discrete number and unit of measurement
☐	☒	A statement on whether measurements were taken from distinct samples or whether the same sample was measured repeatedly
☐	☒	The statistical test(s) used AND whether they are one- or two-sided<br>*Only common tests should be described solely by name; describe more complex techniques in the Methods section.*
☒	☐	A description of all covariates tested
☒	☐	A description of any assumptions or corrections, such as tests of normality and adjustment for multiple comparisons
☐	☒	A full description of the statistical parameters including central tendency (e.g. means) or other basic estimates (e.g. regression coefficient) AND variation (e.g. standard deviation) or associated estimates of uncertainty (e.g. confidence intervals)
☐	☒	For null hypothesis testing, the test statistic (e.g. *F*, *t*, *r*) with confidence intervals, effect sizes, degrees of freedom and *P* value noted<br>*Give P values as exact values whenever suitable.*
☒	☐	For Bayesian analysis, information on the choice of priors and Markov chain Monte Carlo settings
☒	☐	For hierarchical and complex designs, identification of the appropriate level for tests and full reporting of outcomes
☒	☐	Estimates of effect sizes (e.g. Cohen's *d*, Pearson's *r*), indicating how they were calculated

Our web collection on statistics for biologists contains articles on many of the points above.

Software and code

Policy information about availability of computer code

Data collection	Raw reads of RNA-seq data were collected by BGI Japan Corporation using the Illumina HiSeq 2500 platform.
Data analysis	RNA-seq read data were mapped to the Ppolytes.v1.0.0. transcriptome using the analytical software Bowtie 2. Genes whose expression varied under the control of dsx-H were identified by comparing gene expression levels from the mapping status, using analytical software DESeq.

For manuscripts utilizing custom algorithms or software that are central to the research but not yet described in published literature, software must be made available to editors/reviewers. We strongly encourage code deposition in a community repository (e.g. GitHub). See the Nature Research guidelines for submitting code & software for further information.

Data

Policy information about availability of data

All manuscripts must include a data availability statement. This statement should provide the following information, where applicable:

- Accession codes, unique identifiers, or web links for publicly available datasets
- A list of figures that have associated raw data
- A description of any restrictions on data availability

Short-read archive for the P. polytes RNA sequences accession ID, DRR140179 – DRR140184. Wnt1/6 nucleotide sequences were obtained from the following database for each species: Bombyx mori (Wnt1:NP_001037315.1, Wnt6:XP_012548361.1) (KAIKObase: http://sgp.dna.affrc.go.jp/KAIKObase/), Danaus plexippus (Wnt1:EHJ69660.1, Wnt6:EHJ69658.1) (Monarchbase: http://monarchbase.umassmed.edu), Papilio polytes (Wnt1:PpolytesGene0008023, Wnt6:PpolytesGene0008021) and Papilio xuthus (Wnt1:PxuthusGene0002332, Wnt6:PxuthusGene0002330) (PapilioBase: http://papilio.bio.titech.ac.jp/), Papilio machaon (Wnt1: KPJ11870.1, Wnt6: XP_014362722.1) (RefSeq database: http://lepbase.org).

Field-specific reporting

Please select the one below that is the best fit for your research. If you are not sure, read the appropriate sections before making your selection.

☒ Life sciences ☐ Behavioural & social sciences ☐ Ecological, evolutionary & environmental sciences

For a reference copy of the document with all sections, see nature.com/documents/nr-reporting-summary-flat.pdf

Life sciences study design

All studies must disclose on these points even when the disclosure is negative.

Sample size	Except for RNA-seq analysis, all the experiments were performed more than 3 biological replicates and standard deviations were calculated from those data.
Data exclusions	No data were excluded intentionally.
Replication	Except for RNA-seq analysis, all the experiments were repeated more than three times independently.
Randomization	Animals were assigned randomly to experimental and control groups.
Blinding	The data presented did not require the use of blinding.

Reporting for specific materials, systems and methods

We require information from authors about some types of materials, experimental systems and methods used in many studies. Here, indicate whether each material, system or method listed is relevant to your study. If you are not sure if a list item applies to your research, read the appropriate section before selecting a response.

Materials & experimental systems

n/a	Involved in the study	
☒	☐	Antibodies
☒	☐	Eukaryotic cell lines
☒	☐	Palaeontology
☐	☒	Animals and other organisms
☒	☐	Human research participants
☒	☐	Clinical data

Methods

n/a	Involved in the study	
☒	☐	ChIP-seq
☒	☐	Flow cytometry
☒	☐	MRI-based neuroimaging

Animals and other organisms

Policy information about studies involving animals; ARRIVE guidelines recommended for reporting animal research

Laboratory animals	N/A
Wild animals	Wild butterfly females were purchased from Chokan-kabira (Okinawa, Japan).
Field-collected samples	N/A
Ethics oversight	No ethical approval

Note that full information on the approval of the study protocol must also be provided in the manuscript.
