# Supplementary figures and images for: The mimetic wing pattern of Papilio polytes butterflies is regulated by a doublesex-orchestrated gene network
Source: Commun Biol. 2019 Jul 10;2:257. doi: 10.1038/s42003-019-0510-7 (PMC6620351; doi:10.1038/s42003-019-0510-7)

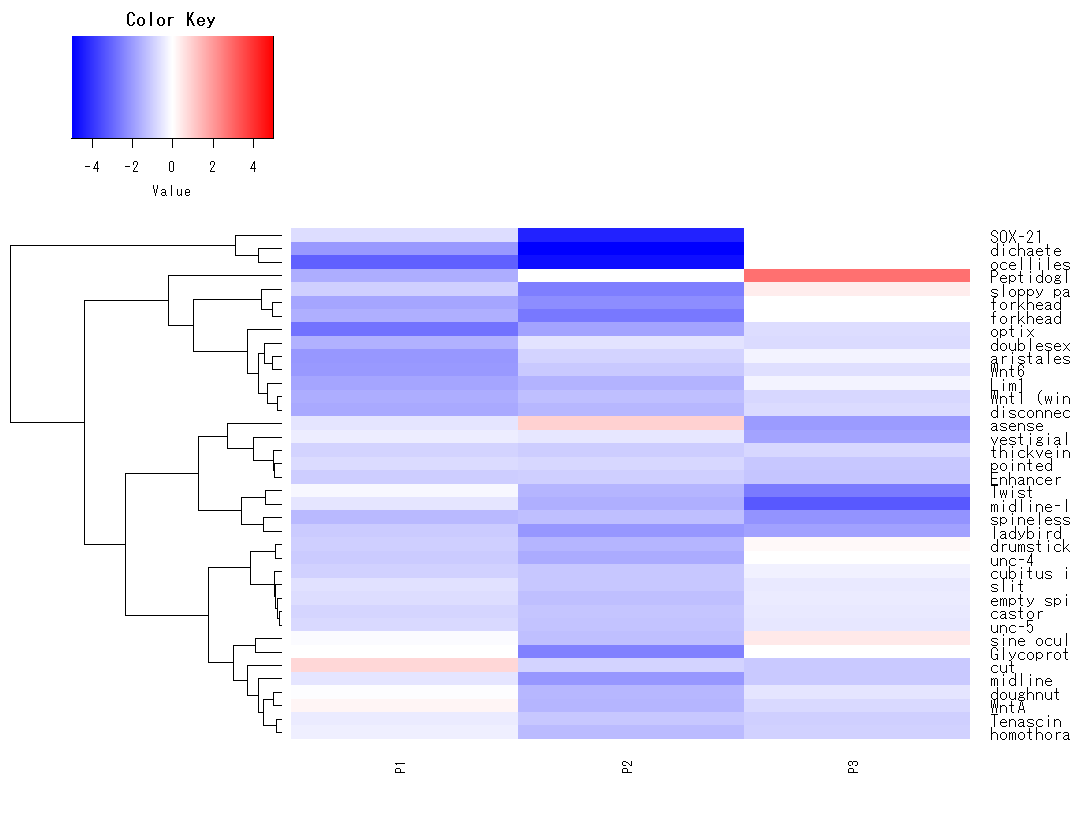

Supplement: Supplementary file 7 — Supplementary Data 4 [file 42003_2019_510_MOESM7_ESM.tif]
